# Human ancient DNA analyses reveal the high burden of tuberculosis in Europeans over the last 2,000 years

**DOI:** 10.1016/j.ajhg.2021.02.009

**Published:** 2021-03-04

**Authors:** Gaspard Kerner, Guillaume Laval, Etienne Patin, Stéphanie Boisson-Dupuis, Laurent Abel, Jean-Laurent Casanova, Lluis Quintana-Murci

**Affiliations:** 1 Human Evolutionary Genetics Unit, Institut Pasteur, UMR2000, CNRS, 75015 Paris, France; 2 Laboratory of Human Genetics of Infectious Diseases, Necker Branch, INSERM UMR 1163, Necker Hospital for Sick Children, 75015 Paris, France; 3 Paris University, Imagine Institute, 75015 Paris, France; 4 St. Giles Laboratory of Human Genetics of Infectious Diseases, Rockefeller Branch, Rockefeller University, New York, NY 10065, USA; 5 Howard Hughes Medical Institute, New York, NY 10065, USA; 6 Chair of Human Genomics and Evolution, Collège de France, 75005 Paris, France

**Keywords:** tuberculosis, genetics, human, evolution, ancient DNA, Europeans, natural selection, disease, Approximate Bayesian Computation, Mycobacterium tuberculosis

## Abstract

Tuberculosis (TB), usually caused by *Mycobacterium tuberculosis* bacteria, is the first cause of death from an infectious disease at the worldwide scale, yet the mode and tempo of TB pressure on humans remain unknown. The recent discovery that homozygotes for the P1104A polymorphism of *TYK2* are at higher risk to develop clinical forms of TB provided the first evidence of a common, monogenic predisposition to TB, offering a unique opportunity to inform on human co-evolution with a deadly pathogen. Here, we investigate the history of human exposure to TB by determining the evolutionary trajectory of the *TYK2* P1104A variant in Europe, where TB is considered to be the deadliest documented infectious disease. Leveraging a large dataset of 1,013 ancient human genomes and using an approximate Bayesian computation approach, we find that the P1104A variant originated in the common ancestors of West Eurasians ∼30,000 years ago. Furthermore, we show that, following large-scale population movements of Anatolian Neolithic farmers and Eurasian steppe herders into Europe, P1104A has markedly fluctuated in frequency over the last 10,000 years of European history, with a dramatic decrease in frequency after the Bronze Age. Our analyses indicate that such a frequency drop is attributable to strong negative selection starting ∼2,000 years ago, with a relative fitness reduction on homozygotes of 20%, among the highest in the human genome. Together, our results provide genetic evidence that TB has imposed a heavy burden on European health over the last two millennia.

## Main text

Infectious diseases have been the leading cause of mortality since the origin of modern humans in Africa and throughout their subsequent dispersals around the world.^1^, ^2^, ^3^, ^4^, ^5^ Tuberculosis (TB [MIM: 607948]) is considered to be the deadliest infection of the common era, with more than one billion deaths over the last 2,000 years,^6^, ^7^, ^8^ and still responsible for more than 1.5 million deaths annually according to the WHO. The human genetic basis of TB susceptibility has remained elusive until the turn of the 21^st^ century, when two rare inborn errors of immunity, autosomal-recessive interleukin-12 receptor b1 (IL-12Rb1) and tyrosine kinase 2 (TYK2) deficiencies, were identified in children with severe TB.^9^^,^^10^ It was only in 2018 that the first common, monogenic predisposition to TB was identified. Homozygotes for the *TYK2* (MIM: 611521) P1104A polymorphism (rs34536443) were found to be at higher risk of developing clinical forms of TB, due to the selective disruption of IL-23-dependent antimycobacterial IFN-g immunity, underlying a recessive trait.^11^ A subsequent study revealed an enrichment in P1104A homozygotes among TB cases of a case-control cohort from the United Kingdom, where the allele is most prevalent today (4%).^7^ The frequency of P1104A, together with its high penetrance for TB in the homozygous state (>0.8),^11^ suggests that about 1/600 British individuals would develop TB during their lifetime because of the mutation, if TB were still highly endemic in Europe.

Pathogen-imposed selective pressures have been paramount during human evolution.^2^^,^^4^^,^^5^ Over the last decade, population genetic studies have documented strong, distinct selection signatures among host defense genes, helping to delineate immunological mechanisms of major importance,^12^ and supporting the notion that microbes have had an overwhelming impact on human genome diversity.^4^^,^^5^ While several studies have provided insight into the periods when malaria has exerted pressure on humans,^13^, ^14^, ^15^, ^16^, ^17^ little is known about the historical burden of other infectious diseases associated with past epidemics. Yet, TB appears to have been more lethal than malaria in the common era,^6^ making it a stronger selective pressure in endemic regions. Recent evidence based on mycobacterial ancient DNA (aDNA) suggests a Holocene dispersal of *M. tuberculosis* <6,000 years ago (ya),^18^^,^^19^ a time frame that coincides with the growth of agricultural communities and anthropogenic environmental changes, which may have favored infectious disease transmission.^20^

To investigate the historical burden of TB in humans, we sought to reconstruct the evolutionary history of the *TYK2* P1104A variant. Indeed, this mutation, in the homozygous state, underlies the only known common, monogenic predisposition to TB.^7^^,^^11^ Moreover, *TYK2* P1104A does not affect the risk for other infectious diseases except, to a milder degree, rare cases of infection by environmental mycobacteria in otherwise healthy individuals.^11^ Whereas disease-protective variants may rapidly increase in frequency owing to positive Darwinian selection, disease-risk alleles are expected to evolve under strong negative selection and be gradually purged from the population. Because negatively selected variants have become rare, very rare, or even extinct, they are harder to study using genetic data from modern human populations. However, with the increasing availability of genomes from ancient individuals, direct measurements of the intensity of selection are now possible, as significant increases or decreases of allele frequencies can be captured with aDNA from time transects.^21^ Thus, the study of the P1104A variant offers an unprecedented opportunity to shed light on the evolutionary history of a deadly human disease such as TB. Of note, P1104A homozygotes have also been shown to enjoy from a protective effect against various autoimmune and inflammatory diseases.^22^^,^^23^ While this effect could have provided a fitness advantage opposed to that attributable to TB infection, the general late onset manifestation of autoimmune and inflammatory disorders makes unlikely the occurrence of a large counteractive effect.

**Figure 1:**
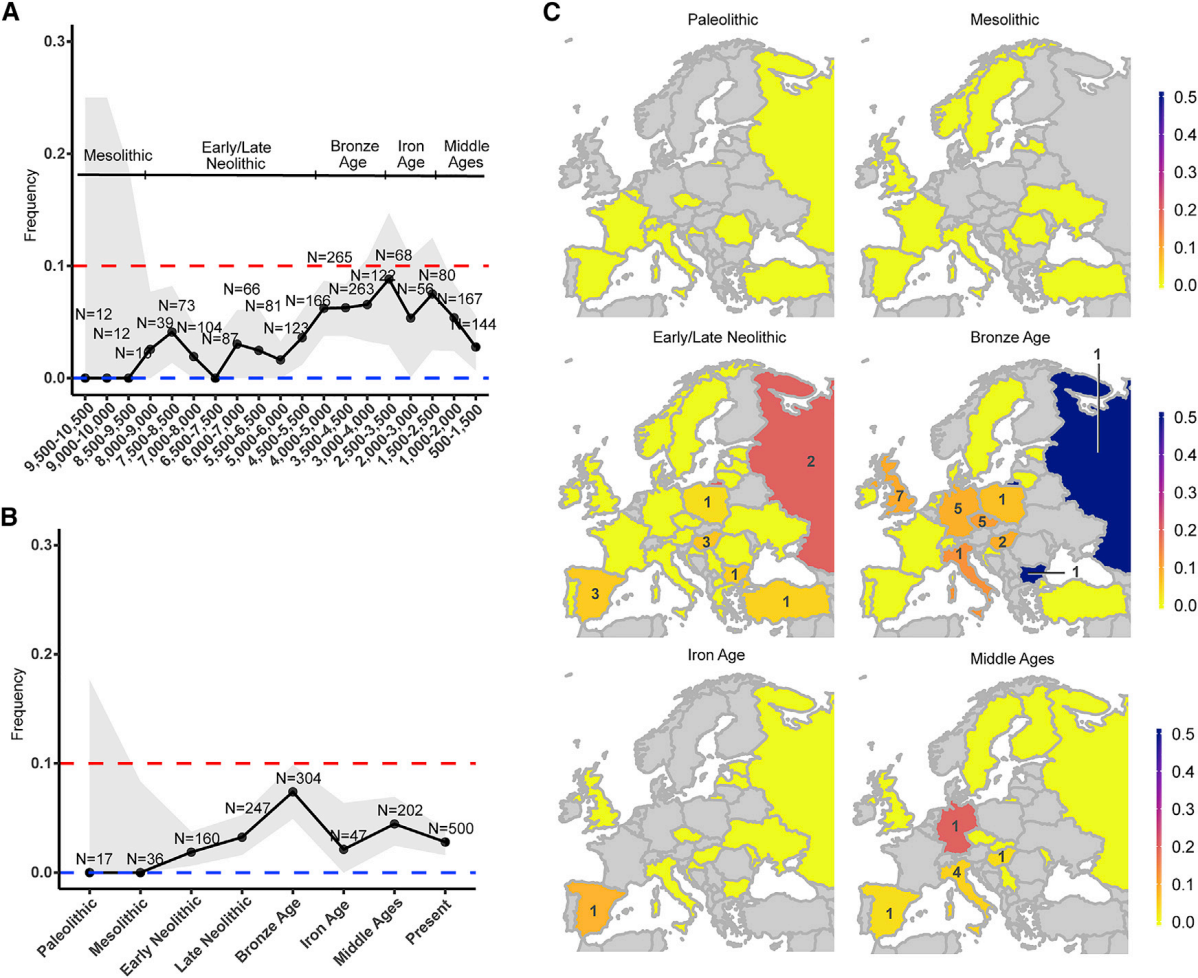
Evolutionary history of the TB-associated *TYK2* P1104A variant (A and B) European frequency trajectory for the *TYK2* P1104A variant over the last 10,000 years for (A) bins of 1,000 years and sliding windows of 500 years or (B) seven (pre-) historical European epochs and current times. The red and blue horizontal dashed lines indicate a frequency of 10% and 0%, respectively. Uncertainty of the frequency estimation is indicated by a gray colored area, representing the normal approximation of the 95% binomial proportion CI. Large uncertainty for older times is due to small sample sizes. For each bin, at least one carrier was assumed to obtain uncertainty estimates. (C) Geographical distribution of the *TYK2* P1104A allele by country (using today’s political borders), across all defined epochs. Colors indicate frequency estimations by country, from 0 (yellow) to 0.5 (blue). Grey indicates unavailable data. Number of P1104A carriers is indicated with its respective number on each country. Sample sizes for countries with non-zero counts (Table S1) are the following: Early/Late Neolithic: Austria (n = 7), Bulgaria (n = 21), Croatia (n = 10), Czech Republic (n = 8), Denmark (n = 1), Estonia (n = 1), France (n = 4), Germany (n = 13), Greece (n = 9), Hungary (n = 51), Ireland (n = 4), Italy (n = 11), Latvia (n = 20), Lithuania (n = 8), Luxembourg (n = 1), Macedonia (n = 1), Norway (n = 1), Poland (n = 32), Portugal (n = 11), Romania (n = 3), Russia (n = 10), Serbia (n = 14), Spain (n = 57), Sweden (n = 11), Turkey (n = 22), UK (n = 46), Ukraine (n = 27); Bronze Age: Bulgaria (n = 2), Croatia (n = 2), Czech Republic (n = 46), Denmark (n = 2), Estonia (n = 7), France (n = 6), Germany (n = 58), Hungary (n = 17), Ireland (n = 1), Italy (n = 8), Lithuania (n = 4), the Netherlands (n = 10), Poland (n = 15), Portugal (n = 2), Russia (n = 2), Spain (n = 33), Sweden (n = 7), Switzerland (n = 1), Turkey (n = 5), UK (n = 75); Iron Age: Bulgaria (n = 1), Croatia (n = 1), Czech Republic (n = 1), Estonia (n = 3), Hungary (n = 5), Italy (n = 6), Latvia (n = 8), Moldova (n = 4), Russia (n = 2), Spain (n = 12), UK (n = 1); Middle Ages: Czech Republic (n = 1), Finland (n = 4), Germany (n = 5), Hungary (n = 30), Iceland (n = 9), Italy (n = 89), Moldova (n = 2), Russia (n = 3), Serbia (n = 1), Slovakia (n = 1), Spain (n = 32), Sweden (n = 13), UK (n = 12).

We therefore examined the frequency trajectory of P1104A over the last 10,000 years of European history, by screening a collection of 1,013 genomes that cover a time transect from the Mesolithic period to the Middle Ages (Figure 1A; Table S1). We partitioned the aDNA data into seven epochs and incorporated data from present-day populations (supplemental material and methods). The P1104A variant, which we found to be the result of a single mutational event (Figure S1), appeared for the first time in our dataset during the early Neolithic ∼8,500 ya in the Anatolian peninsula, and then spread to Central Europe where it remained at frequencies lower than 3% until ∼5,000 ya (Figures 1A–1C). During the Bronze Age, P1104A increased in frequency, reaching its maximum frequency ∼3,000 ya at nearly 10%. After the Iron Age, we observed a strong and consistent decrease in frequency of P1104A, resulting in an average frequency of 2.9% among contemporary Europeans.^24^

We estimated the age of the *TYK2* P1104A mutation (T_age_), tested whether the mutation has been the substrate of natural selection, and inferred the onset (T_onset_) and strength (*s*) of negative selection acting on homozygotes, using an approximate Bayesian computation (ABC) approach^25^ that considers large prior assumptions (T_age_ ∼U[8.5–100,000] ya, T_onset_ ∼U[500–10,000] ya and *s* ∼U[0–1]; supplemental material and methods). We first determined the extent to which our approach could determine the evolutionary model of P1104A that best explains the observed aDNA data, by comparing the fit of the simulated to the observed data (supplemental material and methods). We assumed a validated demographic model for Europeans,^26^ to which we added gene flow from both Near Easterners and Central Asians (Table S2), to account for the large-scale migrations of early farmer populations of the Anatolian plateau and Eurasian steppe populations associated with the Yamnaya culture inferred from aDNA.^27^ In doing so, considering the aforementioned large prior assumptions, we obtained simulated frequency trajectories that closely reproduce that of P1104A, similarly to other genome-wide variants (Figure S2). We also noted a similar, or higher, increase in frequency as that observed for P1104A until the Bronze Age for more than 20% of other aDNA variants within the uncertainty frequency interval of P1104A in the Mesolithic ([0.00–0.10]; Table S3), highlighting the marked impact of the aforementioned migratory events on the frequency of a large fraction of genomic variants, including P1104A. Furthermore, simulated neutral variants closely matched observed frequency distributions of non-coding variants for all epochs (Figure S3), indicating that the demographic model used—present-day Europeans are a mixture of Mesolithic hunter-gatherers, Anatolian Neolithic farmers, and Eastern steppe-related groups^28^^,^^29^—well reproduces the neutral patterns of European diversity.

**Figure 2:**
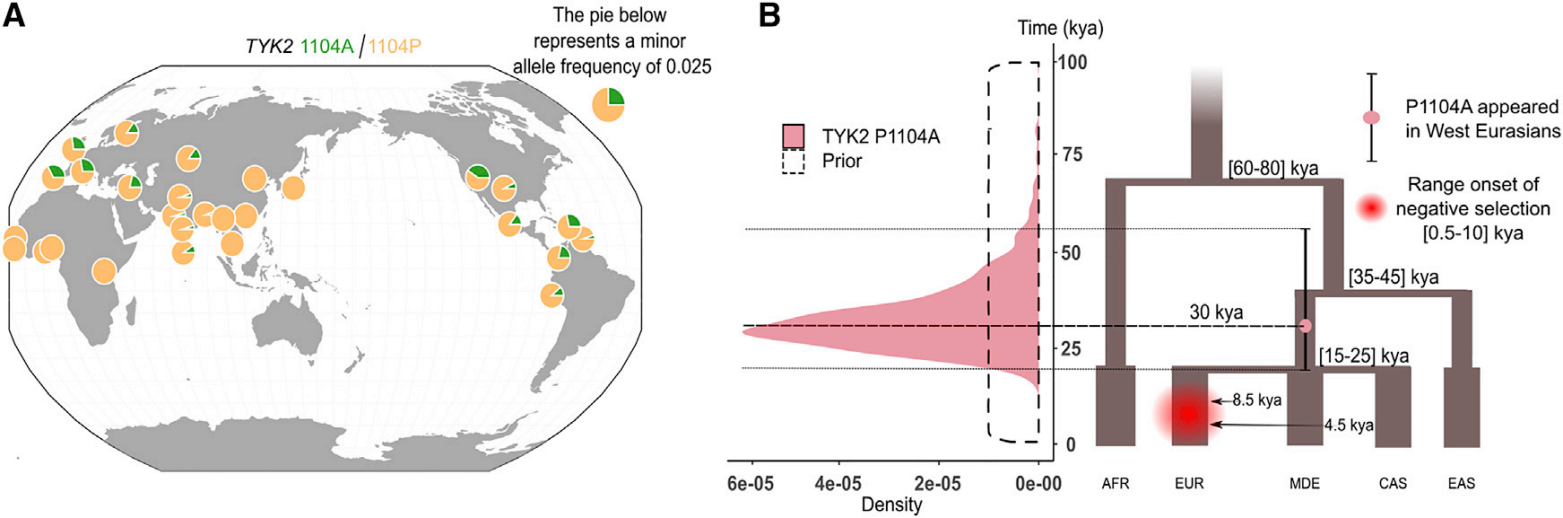
Present-day geographic distribution and age estimation of the *TYK2* P1104A mutation (A) Frequencies from present-day Europeans (EUR; f = 0.03; n = 503), sub-Saharan Africans (AFR; f = 0; n = 504), East Asians (EAS; f = 0; n = 504), Americans (AMR; f = 0.01; n = 347), Middle Easterners (MDE; f = 0.02; n = 163), and Central Asians (CAS; f = 0.01; n = 363) are shown (supplemental material and methods). Figure was built with 1000 Genomes Project data^24^ and modified to include Middle Easterners and Central Asians. The presence of *TYK2* P1104A among American populations from the 1000 Genomes Project reflects recent admixture with Europeans,^24^ with all populations sharing a unique 6 kb-long haplotype around *TYK2* P1104A, although the allele is absent from Native Americans. (B) Left panel: posterior distribution for the age (in thousands of years) of the *TYK2* P1104A mutation, according to the best fitting simulations with variable onset of selection, using 10,000,000 simulations and all available summary statistics. CI boundaries are shown with dashed black lines. Right panel: the proposed demographic model, showing the point estimate for T_age_ (mode = 30 kya, purple red circle) and the 95% confidence interval (black vertical segment across the purple circle) for the age of the mutation.

We then estimated the origin of the *TYK2* P1104A mutation, based on its frequency in K = 12 populations sampled at different epochs, including European aDNA data (Paleolithic, Mesolithic, Early Neolithic, Late Neolithic, Bronze Age, Iron Age, and Middle Ages; supplemental material and methods) and present-day Europeans, Middle Easterners, Central Asians (from 1% to 4%), Sub-Saharan Africans (0%), and East Asians (0%) (Figure 2A; Table S3). We found the age of P1104A to be ∼30,000 years old (mode = 29,182; 95% CI [20,636–57,285]) (Figure 2B; supplemental material and methods), which is consistent with a previous estimate.^30^ Using cross-validation, we found that parameter estimation was accurate across all ages, with 96% of 1,000 estimated 95% CIs including the true simulated value, and also robust to the choice of the summary statistics used (Figures S4A–S4D). While the 95% CI for the age of P1104A overlaps with the divergence time between West and East Eurasians (35–45 kya), the proportion of best-fitting simulated variants originating in the common ancestors of West Eurasians was significantly higher than that of the rest of simulated variants (OR = 7.00, 95% CI [5.70–8.53], p < 10^−10^; Figure 2B; supplemental material and methods). This suggests that P1104A originated in the common ancestors of West Eurasians after the split with East Eurasians, but before the divergence of Europeans, Middle Easterners, and Central Asians. Together, our results provide robust evidence that *TYK2* P1104A appeared during the Upper Paleolithic in West Eurasia, largely predating the estimated emergence of TB in Europe.^18^^,^^19^^,^^31^

We next investigated the evolutionary forces that could explain the frequency decrease of P1104A since the Bronze Age, where the maximum frequency is observed, by simulating frequency trajectories under neutrality (*s* = 0) or negative selection (*s* > 0) (Figure S5A). We found that simulations matching the estimated frequency of P1104A at the end of the Bronze Age explained both the observed aDNA and modern data only if *s* > 0.1. Furthermore, the frequency decrease after the Bronze Age was observed in the trajectories of 25% of the best fitting simulated deleterious variants (*s* ∼U[0–1] and T_onset_ ∼U[500–10,000]; supplemental material and methods), relative to only 1% of the best fitting simulated neutral variants (OR = 33, 95% CI = [5–240], p < 10^−10^; Figure S5B; Table S4). These observations collectively support a history of negative selection driving the evolution of the TB-risk P1104A variant after the Bronze Age.

To quantify the degree of deleteriousness of *TYK2* P1104A during European history, we verified that allele frequency trajectories were informative to assess negative selection, and, encouragingly, we observed a strong positive correlation between drops in allele frequencies and *s* values (Figure S6A). We first hypothesized that negative selection started with the arrival of agriculture in Europe,^20^ a period that includes the upper bound estimation for the most recent common ancestor of the *M. tuberculosis* complex ∼6,000 ya.^18^^,^^19^ However, such an early onset of selection (T_onset_ = 10,000) was clearly rejected by our simulations (Hotelling’s T-squared test p = 5.4 × 10^−4^; Figure S6B; supplemental material and methods; Table S4), as no simulated variants were able to reproduce the frequency increase of P1104A until the Bronze Age. Conversely, when allowing the onset of selection to vary across the last 10,000 years, using the former large priors (T_onset_ ∼U[500–10,000] ya and *s* ∼U[0–1]), our best simulations did not significantly differ from P1104A (i.e., the simulation set was not rejected; Hotelling’s T-squared test p = 0.09) and revealed that scenarios with recent onsets of negative selection were those best fitting the data (Figure S6B).

**Figure 3:**
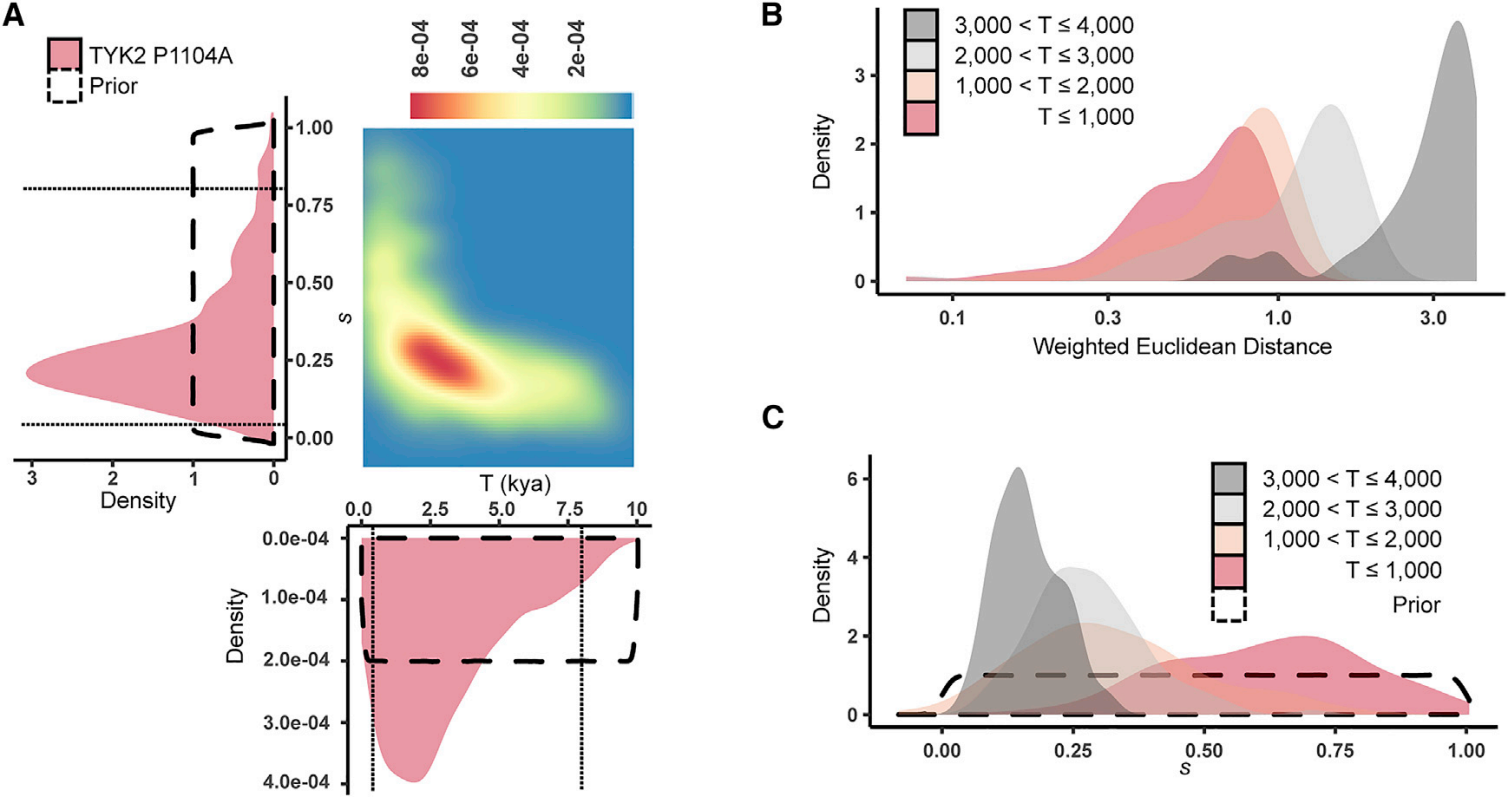
Estimation of the onset and strength of negative selection driving the evolution of *TYK2* P1104A (A) Joint (as a 2D density plot) and individual (as purple densities) posterior distributions for the onset (in thousands of years) and the strength of negative selection (*s*) for *TYK2* P1104A, based on the best fitting simulations with variable onset of selection, using European summary statistics from the Late Neolithic epoch onward (10,000,000 simulations). CI boundaries are shown with dashed black lines. (B) Distribution of the weighted Euclidean distances between the best fitting simulations and the observed data, under the proposed demographic model, for (from right to left) 3,000 < T_onset_≤ 4,000, 2,000 < T_onset_≤ 3,000, 1,000 < T_onset_≤ 2,000, or 500 < T_onset_≤ 1,000. (C) Posterior distributions for the *TYK2* P1104A’s negative selection coefficient, based on the best fitting simulations with variable onset of selection, for the same groups of onsets of selection as in (B) using the same color code.

To explain the strongest frequency increase and decrease for P1104A, we modeled allele frequencies of K = 5 ancient populations (Late Neolithic, Bronze Age, Iron Age, and Middle Ages) and present-day Europeans, and assumed large priors for model parameters (supplemental material and methods, Table S3). We found that negative selection on P1104A homozygous carriers started 1,937 ya (95% CI [500–7,912]), with a selection coefficient of 0.21 (95% CI [0.06–0.82]) (Figures 3A–3C). This onset of selection is consistent with a neutral evolution for the allele until the Bronze Age, suggesting that drift and admixture are sufficient to explain the increase of P1104A frequency until this epoch. These estimations should not be biased owing to read mapping bias of the reference allele in the ancient genome dataset,^32^ given that 1104A is the alternative allele (supplemental material and methods). Furthermore, parameter estimation was found to be robust to the choice of the summary statistics used, with the 95% CIs of the estimates including the true simulated value 93% of the time (Figures S6C and S6D). Although our analysis showed that the more recent the onset of selection was the closer the frequency trajectory estimation was to the empirical data (Figure S6A), the fit was found to be similar within the last ∼2,000 years (Figure 3B), consistent with our estimation. With respect to the selection coefficient, the posterior distributions of *s* were shifted to 1 as T_onset_ became closer to 0, and the general posterior distribution for the strength of negative selection was similar to that of onsets of selection occurring between 1,000 and 3,000 ya (Figures 3A and 3C). Importantly, consistent ABC estimates of the strength and the onset of selection were found when either excluding the Iron Age, i.e., the epoch with smallest sample size (*s* = 0.19; 95% = [0.03–0.83]; T_onset_ = 1,670 ya; 95% CI = [500–8,388] ya) or when using the whole European frequency trajectory, i.e., from the Paleolithic to the present (*s* = 0.21; 95% = [0.04–0.84]; T_onset_ = 1,567 ya; 95% CI = [500–8,367]).

Using the same approach, we estimated the selection coefficient of another mutation, *TYK2* I684S, a missense variant that is neither in linkage disequilibrium with P1104A nor associated with TB risk,^11^ and found values that were compatible with neutrality (*s* = 0.02; 95% CI [0–0.19]; Figures S7A and S7B). Thus, our analyses support the notion that, despite the reported protective effects of P1104A against some immune-related disorders,^22^^,^^23^ TB has exerted pressure on the *TYK2* P1104A variant over the last ∼2,000 years, with a 20% relative fitness reduction for homozygotes at each generation since.

Finally, we sought to apply the same approach to reported pathogenic variants, by cross-matching the ClinVar database^33^ with aDNA variants present in our cohort that fall into the uncertainty range of P1104A in the Bronze Age ([0.04–0.10], Figure 1B). Among the resulting three variants with a “pathogenic” clinical significance annotation, only one (*HFE* C282Y [MIM: 613609]) presents a frequency decrease across the last four epochs. *HFE* C282Y is a known disease-causing variant underlying hemochromatosis, an autosomal-recessive autoimmune disease (HFE1 [MIM: 235200]) that impairs mineral metabolism, which can affect the growth and clearance of intra- and extra-cellular pathogens.^34^ *HFE* C282Y reached its maximum frequency, of nearly 10%, during the Middle Ages and then decreased to its present-day frequency of 4%. Consistent with our expectations, we found a similarly strong selection coefficient of 0.20 (mode = 0.22; 95% CI [0.03–0.76]; Figure S7B), and an onset of negative selection during the Middle Ages (mode = 724 ya; 95% CI [500–7,508]).

**Figure 4:**
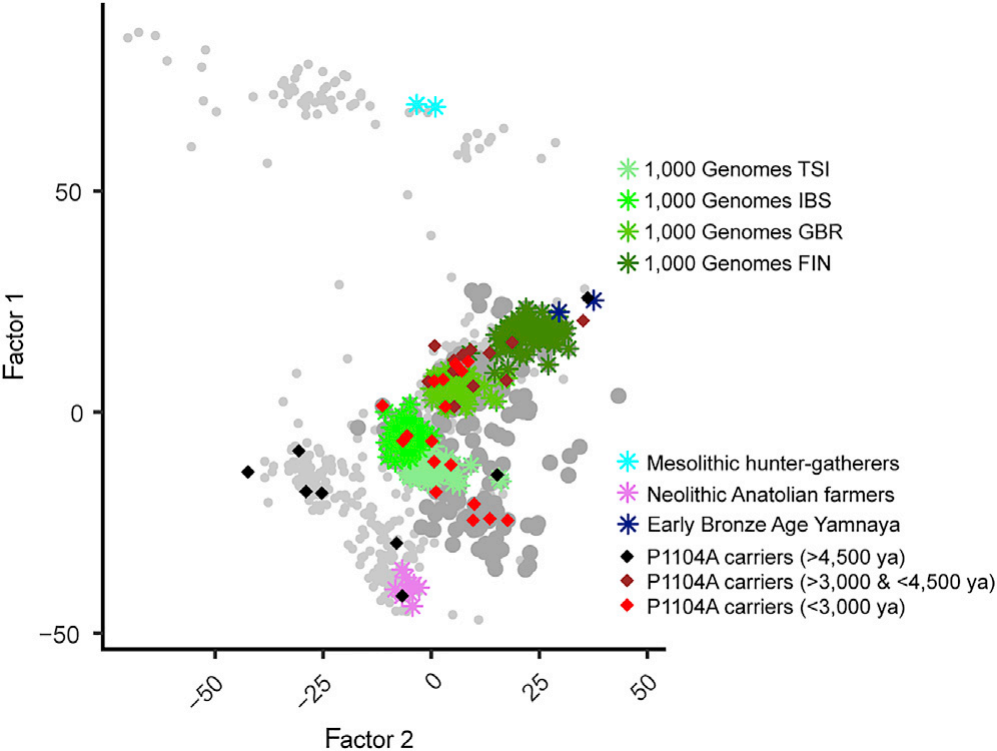
*TYK2* P1104A carriers scatter throughout the genetic diversity of the dataset Factor analysis (Factor 2 versus Factor 1) for 701 high-quality ancient genomes from the full set of 1,013 used in this work, and 363 pseudo-haploid present-day genomes (TSI, IBS, GBR, and FIN, green colors from lighter to darker, respectively) from various European populations from the 1000 Genomes Project (supplemental material and methods). Two Mesolithic hunter-gatherers (cyan), eight Neolithic Anatolian farmers (violet), and two Early Bronze Age individuals associated with the Yamnaya (>80% steppe ancestry) culture (blue) account for the three major ancestries existing in present-day Europeans, which are, in turn, correlated with their respective epochs. P1104A carriers are shown with black (>4,500 ya), brown (>3,000 ya and <4,500 ya), or red (<3,000 ya) diamonds. Other individuals, older (light) or younger (dark) than 3,000 ya, are represented by gray dots.

A potential limitation of our approach, which is inherent to most aDNA studies, is genetic discontinuity due to large population replacements or to sampling bias for geographical locations.^35^ For example, different sampling proportions from northern and southern Europeans across epochs may result in genetic discontinuity in our dataset, given that the former present higher Eastern steppe ancestry than the latter after the Bronze Age.^36^ We thus repeated our ABC setup for northern and southern Europeans using a geographical division,^37^ designed to distinguish high and low levels of Steppe ancestry (Figure S8). Despite much lower sample sizes, we found evidence for negative selection in both northern (*s* = 0.24; 95% CI: [0.02–0.87]) and southern (*s* = 0.13; 95% CI: [1.6 × 10^−4^–0.81]) European homozygotes, with a slightly left-shifted posterior distribution in southern Europe, where the sample size is more limited (Figure S9). We also found, using factor analysis,^38^ that P1104A carriers scattered throughout European sub-structured populations, across all epochs after its introduction to Europe (Figure 4).

In addition, ancestry proportions were similar between P1104A carriers and the rest of the dataset at each epoch (Table S1). Notably, the observed ancestry shift between Bronze Age and present-day samples (from 0.29 to 0.36 for the whole dataset [Table S3], representing a 24% relative increase, and from 0.23 to 0.39 for P1104A carriers [Table S1]) does not, on its own, explain the frequency decline of the allele after the Bronze Age (from 0.074 to 0.029, representing a 61% relative decrease). Yet, we performed an ABC estimation accounting for ancestry variation across epochs (supplemental material and methods). Using the estimated Anatolian ancestry of our dataset at each epoch from the Late Neolithic onward, we estimated very similar values for the strength and onset of negative selection for *TYK2* P1104A at the pan-European level (*s* = 0.27; 95% CI: [0.08–0.93]; T_onset_ = 2,045 ya; 95% CI [500–8,690]; Figures S10A and S10B). Similarly, we found comparable estimations for northern and southern Europeans (*s* = 0.26; 95% CI [0.06–0.83]; T_onset_ = 1,046 ya; 95% CI [500–6,934]; Figures S10C and S10D; and *s* = 0.24; 95% CI [0.02–0.85]; T_onset_ = 3,229 ya; 95% CI [500–8,963]; Figures S10E and S10F, respectively). Conversely, we found no evidence of selection for *TYK2* I684S (*s* = 0.02; 95% CI: [0–0.69]), as expected, and a weaker signal of negative selection for *HFE* C282Y (*s* = 0.12; 95% CI: [0–0.76]). Collectively, these findings suggest that the observed frequency drop of P1104A after the Bronze Age is not due to major geographical and/or temporal differences in ancestry components in our aDNA dataset, but instead to the action of natural selection. Moreover, when re-estimating the age of P1104A without modern data from Middle Easterners and Central Asians, as they are not entirely representative of ancestral Anatolian farmers and steppe herders, respectively,^39^^,^^40^ we obtained almost identical results (mode = 30,303 ya; 95% CI [23,113–60,273]).

In this attempt to define the mode and tempo of TB pressure on Europeans, we found that the only common variant known to underlie monogenic predisposition to TB has evolved under strong negative selection in Europe after the Iron Age. In doing so, we provide population genetic evidence for the high burden of TB in Europeans over the last two millennia, in line with the dating of *M. tuberculosis* lineage 4 at 1,943 ya^41^ and of strains found in 18^th^ century Hungarian mummies at 1,604 ya, or in mummified remains of the 17^th^ century Bishop Peder Winstrup of Lund between 929 and 2,084 ya.^19^^,^^31^ Notably, the TB-associated mutation ranks among the top 2.7% of variants, present in the studied capture array, with similar frequencies in the Bronze Age (0.04–0.10) that have decreased the most since this period (Table S3; supplemental material and methods). Such variants might also include targets of negative selection (Table S5). A selection coefficient of 0.20 would entail >2,500,000 cumulative deaths over the last 2,000 years due to P1104A homozygosity, representing 1%–2% of all TB-related deaths in the 19^th^ century Europe (Figure S11). This figure is consistent with a previous estimation of 1% of TB cases due to the at-risk genotype among present-day Europeans.^7^ We anticipate that the same population genetics framework could be used to delineate other human genetic variants, of yet unknown function, that have drastically decreased or increased in frequency across time due to microbial pressure. Thus, adopting an evolutionary approach represents a promising alternative to investigate the genetic sources of present-day disparities, between individuals and populations, in susceptibility to infection.

## Data and code availability

Pseudo-haploid ancient and modern genome data are available at https://reich.hms.harvard.edu/allen-ancient-dna-resource-aadr-downloadable-genotypes-present-day-and-ancient-dna-data (V42.4: March 1, 2020 release). Code to perform ABC estimations from simulated frequency data are available at https://github.com/h-e-g/SLiM_aDNA_selection.

## Declaration of Interests

The authors declare no competing interests.

## Web resources

OMIM, https://www.omim.org/

## Supplemental Information

Document S1. Supplemental material and methods, Figures S1–S11, and Tables S2–S5

Table S1. Collection of 1,013 ancient genomes covering a time transect from the Mesolithic period to the Middle Ages

Document S2. Article plus supplemental information
