# Supplementary material for: Human ancient DNA analyses reveal the high burden of tuberculosis in Europeans over the last 2,000 years
Source: Am J Hum Genet. 2021 Mar 4;108(3):517–24. doi: 10.1016/j.ajhg.2021.02.009 (PMC8008489; doi:10.1016/j.ajhg.2021.02.009)
Supplement: Document S1. Supplemental material and methods, Figures S1–S11, and Tables S2–S5 [file mmc1.pdf]

The American Journal of Human Genetics, Volume *108*

Supplemental data

Human ancient DNA analyses reveal the high burden of tuberculosis in Europeans over the last 2,000 years

Gaspard Kerner, Guillaume Laval, Etienne Patin, Stéphanie Boisson-Dupuis, Laurent Abel, Jean-Laurent Casanova, and Lluis Quintana-Murci

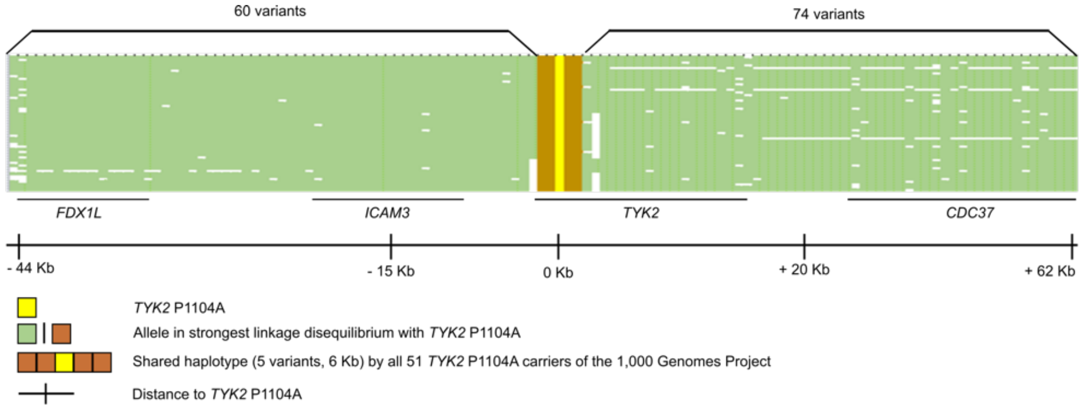

Figure S1. Founder effect for *TYK2* P1104A. Haplotypes (rows) for all 51 European (N=29), European-American (N=14), South Asian (N=6) and African-American (N=2) carriers of the *TYK2* P1104A allele of the 1,000 Genomes Project.[1] Variants (columns) outside a 6 Kb region around *TYK2* P1104A are colored according to whether the individual carries (green) or not (white) the allele in strongest linkage disequilibrium with *TYK2* P1104A for all biallelic sites with >1% frequency in 1,000 Genomes Project. All 51 heterozygous carriers of *TYK2* P1104A share a 6 kb-long haplotype composed of the variant itself, two variants upstream and two downstream it, which are the closest variants to *TYK2* P1104A, suggesting a worldwide founder effect.

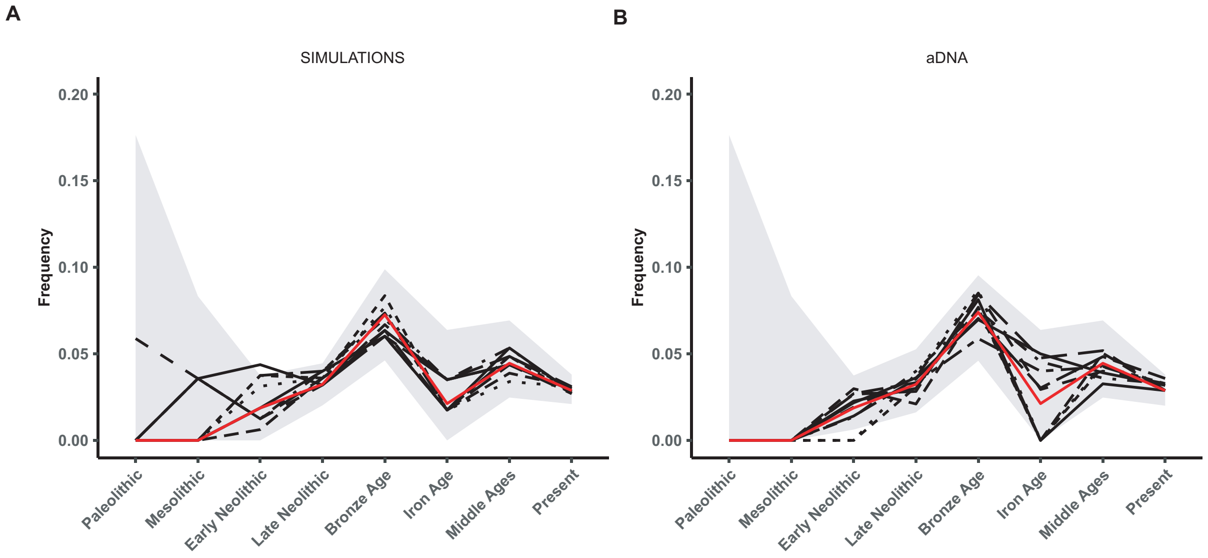

Figure S2. Top ten simulated or real aDNA variants best fitting the *TYK2* P1104A frequency trajectory. Top ten variants best fitting the observed data for (**A)** simulated variants under the proposed demographic model, with (uniformly distributed T_{onset} <10,000) or without negative selection, or (**B)** real aDNA data. The data is based on either 1,000,000 simulations or 1,000,000 randomly sampled aDNA variants. The red trajectory is that of *TYK2* P1104A. Uncertainty of the estimation is represented for *TYK2* P1104A frequencies as in **Figure 1**.

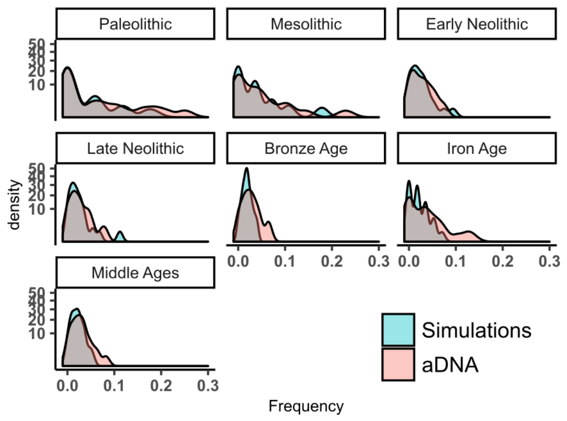

Figure S3. Matching between randomly simulated and real aDNA variants across different epochs. Densities of frequency for 500 randomly sampled simulated neutral or real aDNA variants with 3% frequency among present-day Europeans, for all seven (pre-) historical epochs. The y-axis is in root square scale for better visualization.

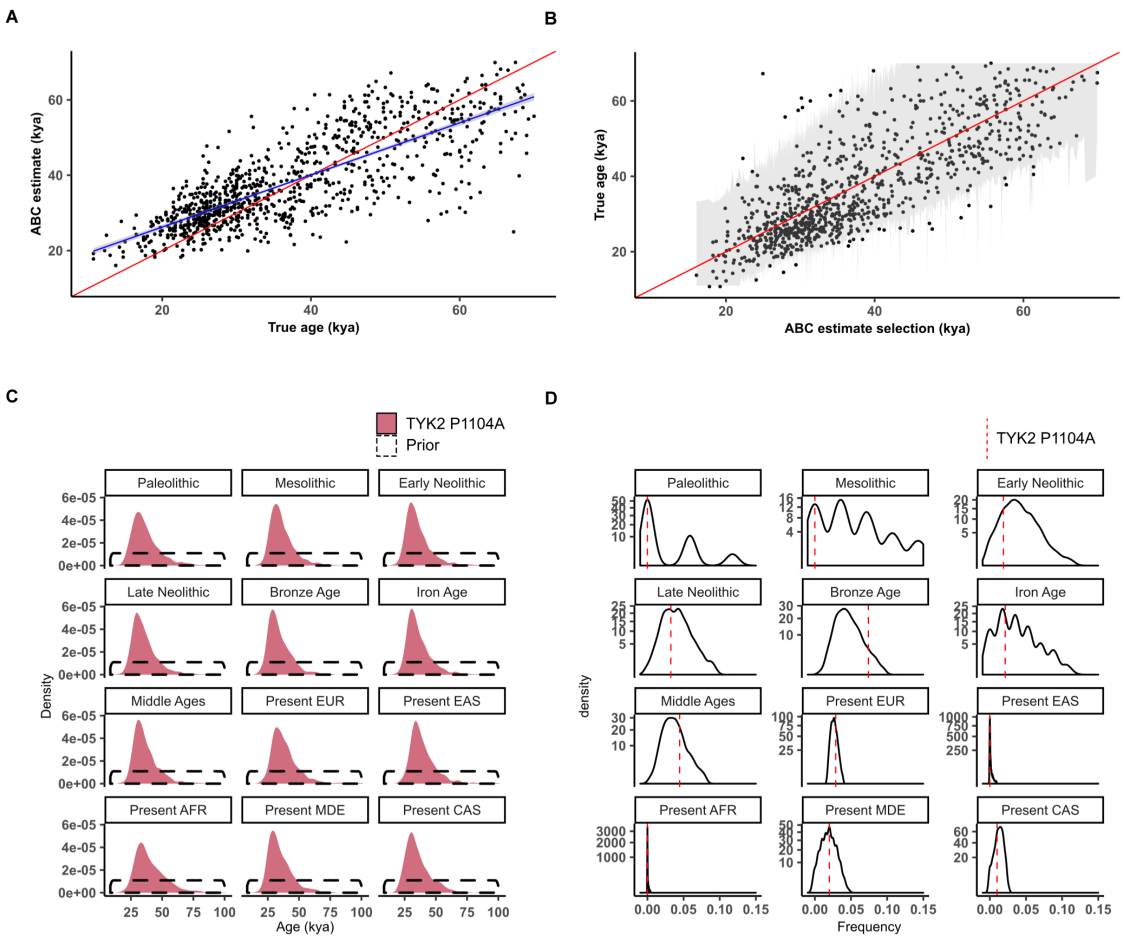

Figure S4. Model accuracy to assess the age of the *TYK2* P1104A mutation. **(A)** Leave-one-out cross validation model for the estimation of age, based on simulations in which the variant exists in present-day Europe, for N=1,000 replicates. The diagonal red line corresponds to the identity function and the blue line is the linear regression estimation on the data. True ages until 70,000 years are represented. True old (>50,000 years) ages are more inaccurately estimated than young (<50,000 years) ages. **(B)** The grey area indicates the range of 95% CIs (up and down bounds) for each ABC estimation of the age of mutation (ranked in ascendant order, *x*-axis). Black dots indicate the true age values for each corresponding ABC estimation, as in (A). For a 95% confidence interval, 96% of the estimated confidence intervals contain the true value. **(C)** Posterior distributions for the age of the *TYK2* P1104A mutation (as in **Figure 2B**), with each panel corresponding to the estimated distribution after removing one summary statistic (label on top) at a time. **(D)** Density distribution for each summary statistic (label on top) using only the simulations best fitting our data with selection and T_{onset} <10,000. Vertical dashed lines indicate the true value for *TYK2* P1104A. The y-axis is in root square scale for better visualization.

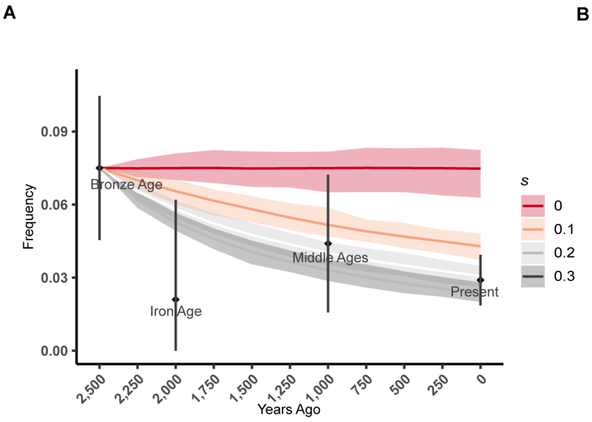

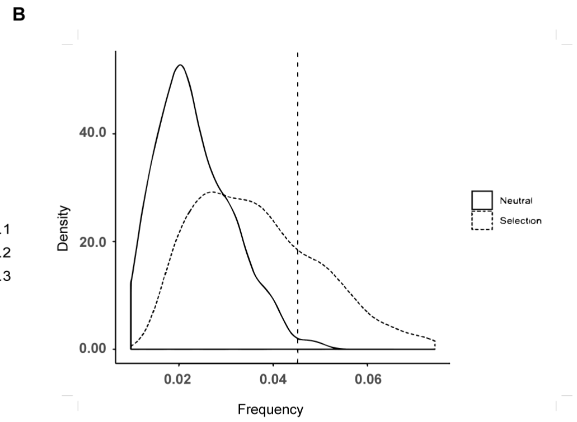

Figure S5. Unmatched frequency trajectory between neutrally-simulated variants and *TYK2* P1104A. **(A)** Average population frequency trajectories of 1,000 simulated variants from the end of the Bronze Age to the present, under the proposed demographic model, with an initial population frequency of 7.5% corresponding to that of P1104A 2,500 ya and a constant effective population sample size of 10,000, assuming neutrality (s = 0) or negative selection (s = 0.1, s = 0.2, s = 0.3). Colored areas indicate the 95% CI of the simulated frequencies at the population level at each generation. Black diamonds represent the P1104A frequency obtained from aDNA at each epoch since the Bronze Age (black bars indicate the confidence interval for the estimated frequency obtained from the aDNA sample, as in **Figure 1B**). **(B)** Distribution of the frequency variation between the Bronze Age and the present in Europe, for neutrally (solid line) or negatively-selected (dotted) simulated variants best fitting the observed data, using 1,000,000 simulations, each. The vertical dashed line represents the real frequency decrease observed for *TYK2* P1104A.

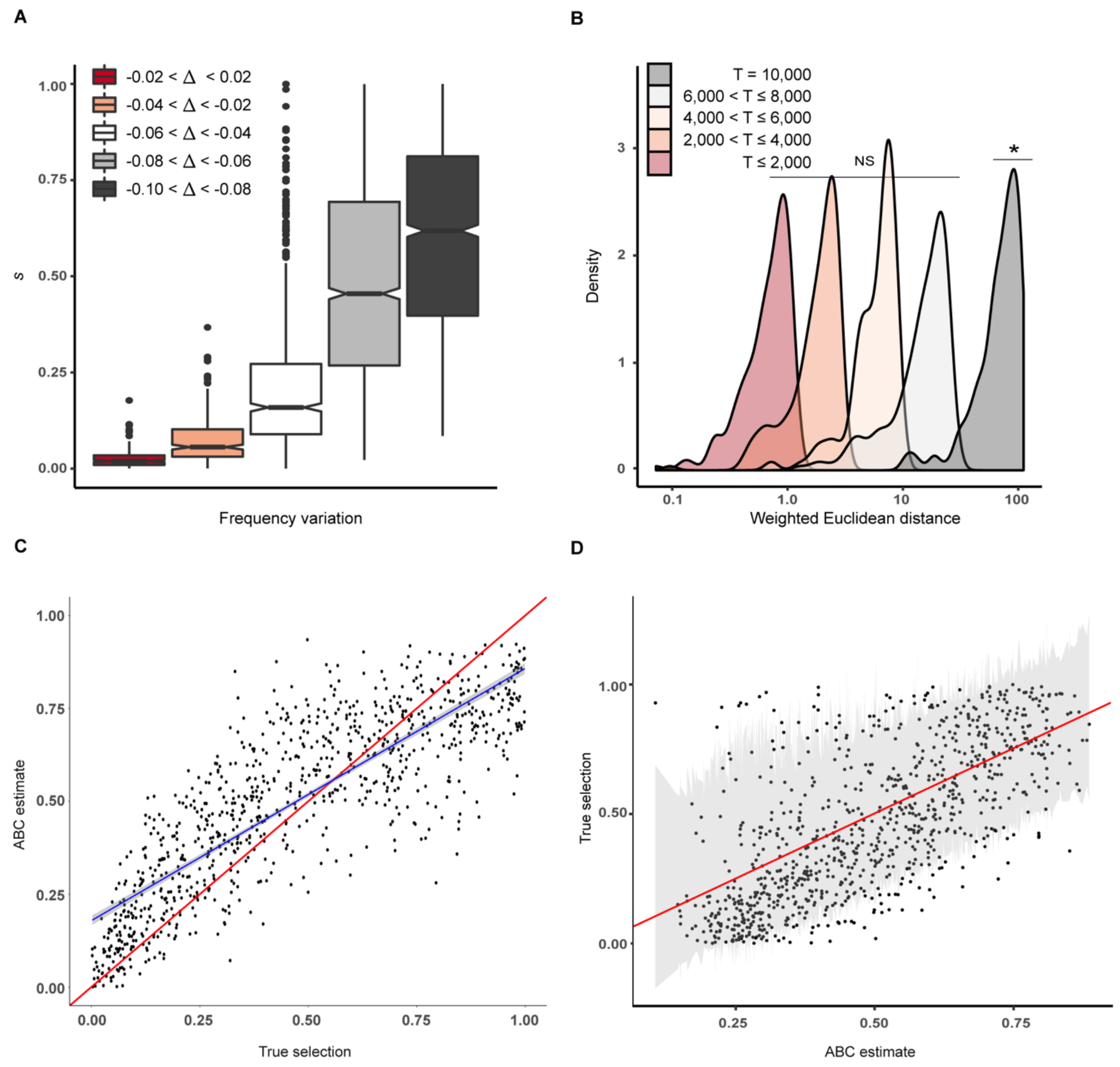

Figure S6. Model accuracy to assess negative selection. **(A)** Distribution of the estimated negative selection coefficient (s), as a function of the absolute variation in frequency (Δ) in a frame of 3,000 years. Δ is negative for variants that decreased in frequency over time. **(B)** Distribution of the weighted Euclidean distances as in **Figure 3B**, with T_{onset} = 10,000 ya (dark grey), 6,000 < $T_{onset} \leq$ 8,000 (light grey), 4,000 < $T_{onset} \leq$ 6,000 (light peach), 2,000 < $T_{onset} \leq$ 4,000 (dark peach), or 250 < $T_{onset} \leq$ 2,000 (red), using European summary statistics from the Late Neolithic epoch onwards. For each T_{onset} interval, 2,000,000 simulations were used. A two-sampled Hotelling's T-squared test was used to assess whether the simulations best fitting our data for the early onset model (T_{onset} = 10,000; $p = 5.4 \times 10^{-4}$; *) or for that with variable onset of selection model (p = 0.09; NS: non-significant) were drawn from the same distribution than that of *TYK2* P1104A. The x-axis is in log-scale for a better visualization. **(C)** Leave-one-out cross validation (N=1,000) for the strength of negative selection, as in **Figure S4A**. The analysis was obtained for simulated variants best fitting the frequency of *TYK2* P1104A at the Bronze age, with T_{onset} <10,000 and 10,000,000 simulations. **(D)** The grey area indicates the range of 95% CIs (up and down bounds) for each ABC estimation of the selection coefficient (ranked in ascendant order, x-axis). Black dots indicate the true values for each corresponding ABC estimation, as in (C). For a 95% confidence interval, 93% of the confidence intervals contain the true value.

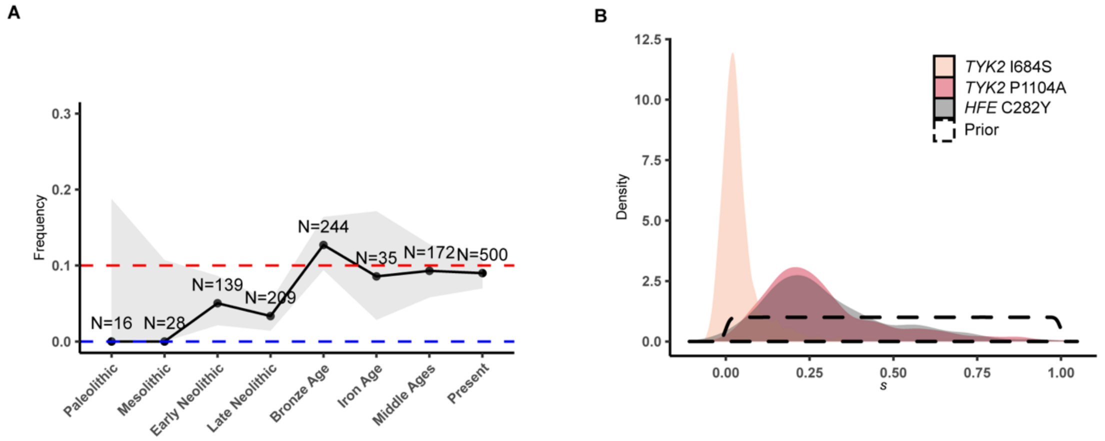

Figure S7. Selection estimations for *TYK2* I684S used for comparative purposes. **(A)** Frequency trajectory for *TYK2* I684S, as in **Figure 1B**. **(B)** Posterior distributions for the strength of negative selection for *TYK2* I684S (beige) and *TYK2* P1104A (red), as in **Figure 3A**.

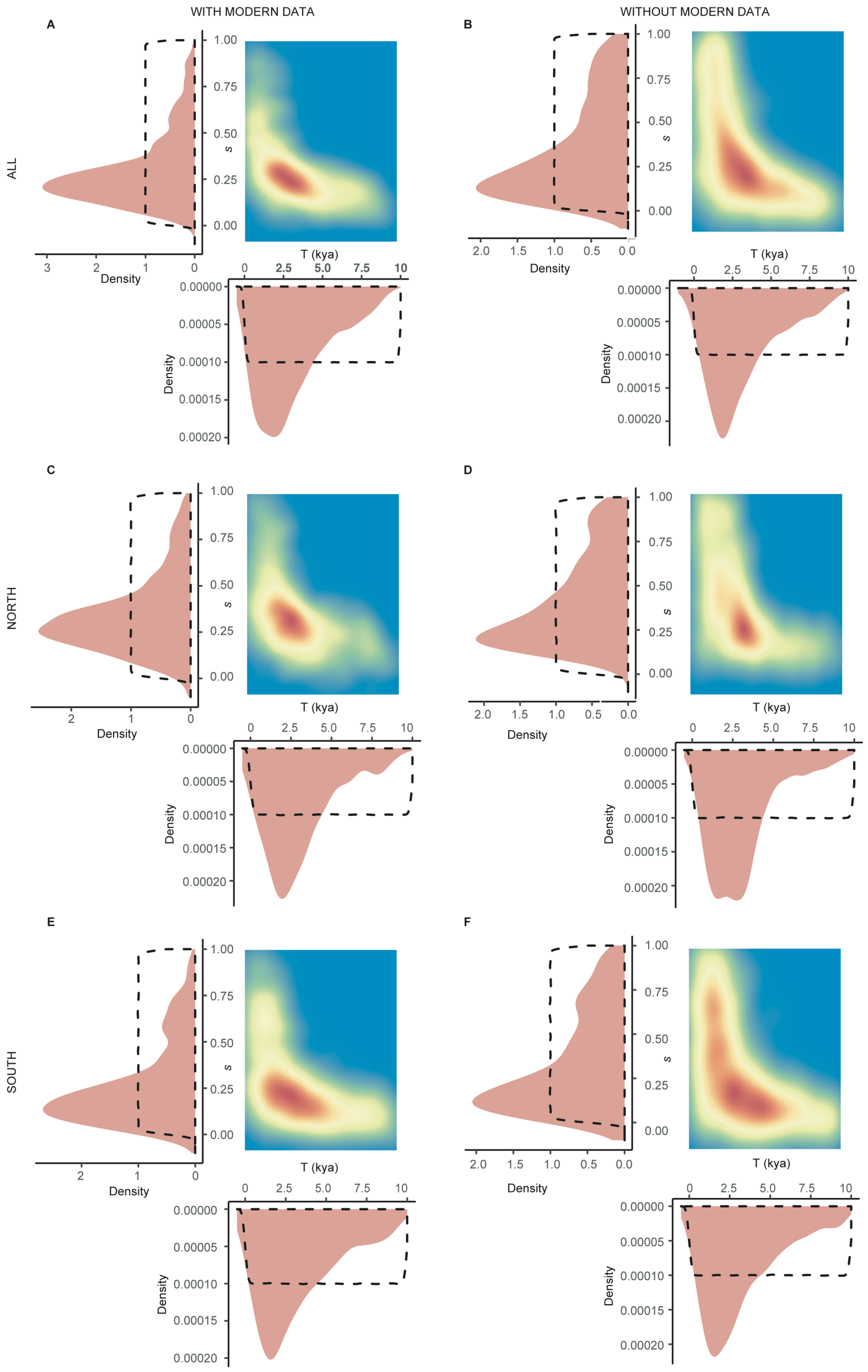

Figure S8. Estimation of the onset and strength of negative selection for *TYK2* P1104A in the case of population stratification or read-bias. Posterior distributions for the negative selection coefficient and the onset of negative selection as in **Figure 3A** for the European population as defined in the main text **(A)** including (*s*: mode = 0.21; 95% CI: [0.06-0.82];) or **(B)** excluding (*s*: mode = 0.13; 95% CI: [0.02-0.94]) modern data, the Northern European population (Supplemental Methods) **(C)** including (*s*: mode = 0.24; 95% CI: [0.02-0.87]) or **(D)** excluding (*s*: mode = 0.21; 95% CI: [0.10-1]) modern data, and the Southern European population (Supplemental Methods) **(E)** including (*s*: mode = 0.13; 95% CI: [1.6×10^{-4}-0.81]) or **(F)** excluding (*s*: mode = 0.11; 95% CI: [0-0.94]) modern data. In all cases considered, we found similar estimations for *s* (higher for northern Europeans, intermediate for the merge of all European populations, and slightly lower for southern Europeans**)**. This suggests that our estimation is not biased due to a systematic read-bias in the aDNA samples.

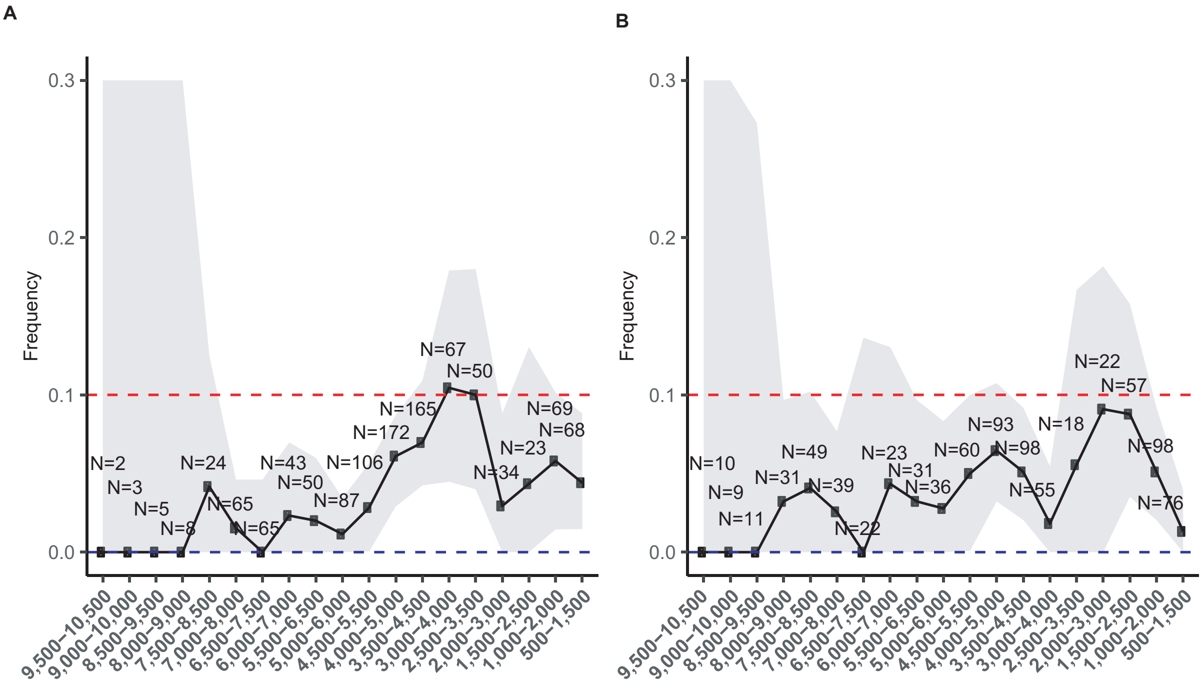

Figure S9. Evolutionary history of the TB-associated *TYK2* P1104A variant in Northern or Southern Europe. **(A)** Northern (with high Steppe-ancestry) or **(B)** Southern **(**low Steppe-ancestry) European frequency trajectories for the *TYK2* P1104A variant over the last 10,000 years for bins of 1,000 years and sliding windows of 500 years as in **Figure 1A**.

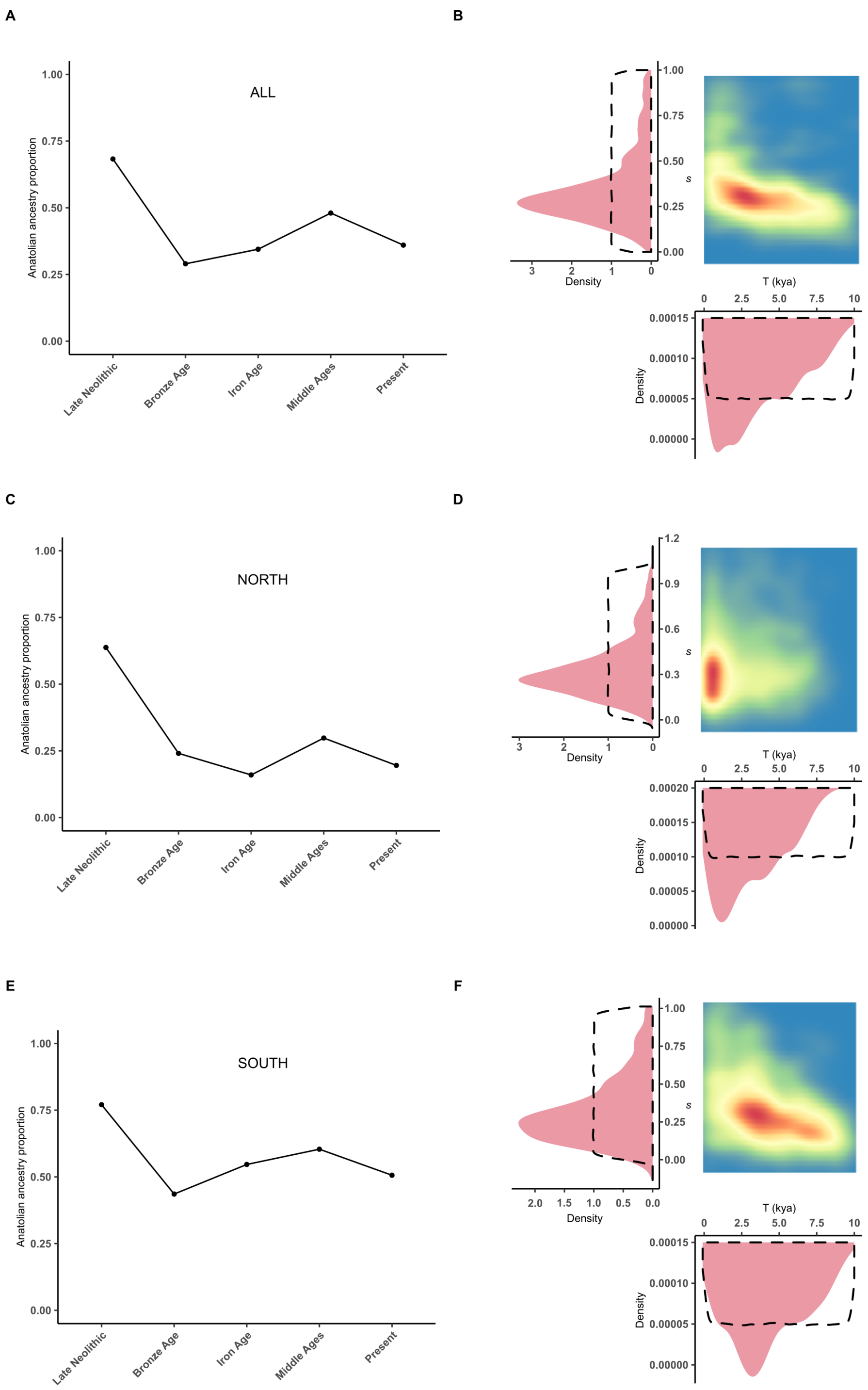

Figure S10. Left part of the figure: Anatolian ancestry trajectory on the basis of **(A)** the full dataset of available aDNA genomes, **(C)** only northern Europeans and **(E)** only southern Europeans. Right part: Estimation of the onset and strength of negative selection for *TYK2* P1104A when accounting for ancestry variations across epochs for **(B)** the full dataset, **(D)** only northern Europeans and **(F)** only southern Europeans. Posterior distributions for the negative selection coefficient and the onset of negative selection are shown as in **Figure 3A**.

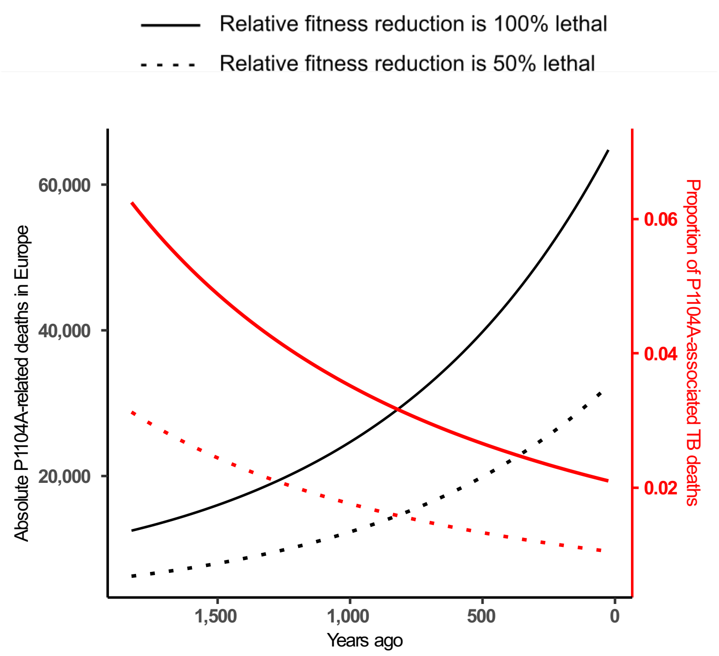

Figure S11. *TYK2* P1104A-related deaths or TB deaths in Europe during the common era. Absolute number of *TYK2* P1104A-related deaths (black) or proportion of TB-related deaths due to *TYK2* P1104A (red), assuming the relative fitness reduction (as estimated by our approach) to be 100% (solid) or 50% (dotted) lethal.

Table S1. Collection of 1,013 ancient genomes covering a time transect from the Mesolithic period to the Middle Ages.

Uploaded as a separate excel file ‘Table_S1.xlsx’

Table S2. Parameters and value ranges to calibrate the updated demographic model

Parameter	Minimum value	Maximum value
Split AFR-NonAFR (*kya*)	60	80
Ne (NonAFR)	2,000	3,000
Split WestNonAFR-EastNonAFR (*kya*)	35	45
Ne (WestNonAFR)	3,000	4,000
Split EUR-Anatolia/Steppe (*kya*)	15	25
Ne (Anatolia/Steppe)	= Ne WestNonAFR	= Ne WestNonAFR
Pulse-Anatolia (*kya*)	8	10
Pulse-Steppe (*kya*)	4	6
Intensity Pulse-Anatolia	0	100
Intensity Pulse-Steppe	0	100

Note: Minimum and maximum values define the accepted range for each parameter. A uniform distribution was used to drawn values from these ranges. *Split* stands for divergence time, *Ne* for effective population size, *Pulse* for the event of instantaneous migration (accounting for x% of migrants of the source population, where x is given by the intensity of the pulse), and *kya* for thousands of years.

Table S3. Values for the empirical summary statistics

Summary Statistic	Value	Uncertainty range[a] (P1104A frequencies)
Paleolithic	0	[0-0.171]
Mesolithic	0	[0-0.081]
Early Neolithic	0.019	[0-0.040]
Late Neolithic	0.032	[0.010-0.054]
Bronze Age	0.076	[0.041-0.107]
Iron Age	0.021	[0-0.062]
Middle Ages	0.045	[0.016-0.074]
Present Europe	0.029	[0.018-0.039]
Present Middle East	0.021	-
Present Central Asia	0.010	-
Present Sub-Saharan Africa	0	-
Present East Asia	0	-
Late Neolithic Anatolian Ancestry	0.683	-
Bronze Age Anatolian Ancestry	0.290	-
Iron Age Anatolian Ancestry	0.345	-
Middle Ages Anatolian Ancestry	0.480	-
Present Europe Anatolian Ancestry	0.360	-

[a] Normal approximation of the 95% CI assuming a binomial distribution for the estimated frequency. For frequencies at 0 we assumed the existence of one carrier to draw CIs, a conservative scenario for the uncertainty range.

Table S4. Simulation sets

	**Neutral**	**Early Onset**	**Full**[a]
T_{age}	$\mathcal{U}$[8.5-100] kya	$\mathcal{U}$[8.5-100] kya	$\mathcal{U}$[8.5-100] kya
s	FIXED, $s = 0$	$\mathcal{U}$[0-1]	$\mathcal{U}$[0-1]
T_{onset}	NA[b]	FIXED, $T_{onset} = 10$ kya	$\mathcal{U}$[0.5-10] kya
# Simulations	1,000,000	1,000,000	10,000,000
Tolerance rate[c]	0.0001	0.0001	0.0001
# Best fitting simulations	100	100	1,000

Note: T_{age}: age of the mutation; s: selection coefficient; T_{onset}: onset of Selection. $\mathcal{U}$ stands for Uniform prior distribution. FIXED stands for fixed value share across all simulations. NA stands for "not applicable".

[a] Simulations underlying the Full set were conducted either accounting or not for ancestry, leading to two sets of 10,000,000 simulations. [b] In the Neutral set of simulations, the onset of selection is undefined since there is no selection. [c] The ABC tolerance rate defines the fraction of simulations that defines the best fitting simulations, i.e., the percent of simulations with the lowest Euclidean distances to the data.

Table S5. Selection estimations for the top 10 variants of Figure S2B.

ID variant	Sel (s)	Sel 95% CI_l	Sel 95% CI_u	Onset	Onset 95% CI_l	Onset 95% CI_u
rs10509135	0.34	0.17	1	1,334	500	8,231
rs10810211	0.21	0.05	0.91	2,746	500	9,665
rs11839433	0.22	0.05	0.90	2,568	500	9,362
rs17065399	0.21	0.05	0.89	2,199	500	9,132
rs75948696	0.30	0.12	0.99	1,626	500	8,635
rs4535615	0.27	0.10	0.97	2,033	500	9,009
rs75379571	0.22	0.07	0.92	2,498	500	9,332
rs7593276	0.22	0.06	0.90	2,156	500	9,005
rs9506920	0.25	0.10	0.95	2,087	500	8,923
rs73664354	0.21	0.06	0.92	2,721	500	8,729

Note: Estimations have been conducted with ABC accounting for ancestry as presented in section ‘Accounting for ancestry in the ABC estimation’.

Supplemental Methods

Ancient DNA and present-day samples

We analyzed 1,013 aDNA genomes (**Table S1)** that: (i) originate from burial sites of western Eurasia (-9< longitude (°) <42.5 and 36< latitude (°) <70.1), (ii) are covered at the *TYK2* P1104 position, and (iii) were retrieved from the V42.4: March 1 2020 release at https://reich.hms.harvard.edu/downloadable-genotypes-present-day-and-ancient-dna-data-compiled-published-papers. Ancient individuals were treated as pseudo-haploid (i.e. carry only one allele) because most samples are low coverage. We manually removed duplicated samples that were explicitly annotated. For many of these samples, 1,233,013 sites were genotyped using an in-solution capture method, which includes the *TYK2* P1104 variant position, while those with whole-genome shotgun sequence data were genotyped at the same set of sites. We also used the *TYK2* P1104A frequencies observed in Europeans, East Asians and Africans from the 1,000 Genomes Project,[1] as well as from 336 Central Asians and 156 Middle Easterners.[2,3]

TYK2 P1104A frequency trajectory

Ancient genomes were grouped by major time periods divided into well-accepted cultural eras: the Paleolithic (51,000 [oldest data in the database] - 12,000 ya), the Mesolithic (12,000 ya - 8,500 ya), the Early (8,500 ya - 6,500 ya) and the Late (6,500 ya - 4,500 ya) Neolithic, the Bronze Age (4,500 ya - 2,800 ya), the Iron Age (2,800 ya - 2,000 ya) and the Middle Ages (2,000 ya - 500 ya). Data was scarce for the Paleolithic (N = 17), the Mesolithic (N = 36) and the Iron Age (N = 47), while estimations for other time periods were obtained from at least 100 aDNA samples each, the Bronze Age being the most covered (N = 304). We also checked for the existence of annotated first-degree familial relationships among the 1,013 ancient genomes of our dataset. We found that the exclusion of all but one individual of each related group (N = 2 exclusions for the Mesolithic, N = 4 for the Early Neolithic, N = 11 for the Late Neolithic, N = 17 for the Bronze Age, N = 0 for the Iron Age and N = 8 for the Middle Ages) had no effects on frequency estimations of P1104A at each epoch, and, more importantly, that no ancient P1104A carriers were related to another individual of the cohort.

Simulated *TYK2* P1104A frequency trajectory

Computer simulations of an allele evolving under a given demographic model were carried out using SLiM 3 (ref.[4]). Because SLiM is a forward-in-time simulator,[4] the computation times, which depend on both the effective population size N and the number of generations t

considered, are large for the model we aimed to simulate. We thus rescaled effective population sizes and times according to N_e/λ and t/λ, with λ = 10 (ref.[4,5]). We assumed a single origin of the *TYK2* P1104A mutation at different times (see section 'Age and origin estimation for the *TYK2* P1104A mutation'). To simulate aDNA, we randomly sampled simulated diploid individuals at generations corresponding to the radiocarbon calibrated estimations for the age of the 1,013 available ancient genomes. For each sampled individual, we then randomly selected an allele to generate pseudo-haploid data, mirroring the observed pseudo-haploid aDNA data used in this study. Additionally, simulated European, East Asian, African, Central Asian and Middle Easterner present-day individuals were randomly drawn at the last generation of the simulated demography, accounting for the sample sizes of the observed data (see section 'Ancient DNA and present-day samples'). The simulated allele frequency trajectory was therefore computed from simulated pseudo-haploid aDNA and diploid modern data, as it was done for the observed data.

Simulated demographic model

The allele frequency trajectory was obtained and assessed under a classical model for which demographic parameters, including divergence times, effective population sizes, migration rates and exponential growth of the three considered populations (ancestral African population, and West and East Eurasians) were available.[6] Because this model neglects major migratory events inferred with aDNA data,[7] we expanded the model to include the arrival of early farmers from the Anatolian peninsula ~8,500 ya and the subsequent migrations associated with Eurasian steppe-related populations ~4,500 ya.[8-14] In the updated model, divergence times between Europeans and Middle Easterners and Central Asians were defined as nuisance parameters (**Table S2**). The range for these parameters were based on recent estimates from whole-genome sequences of individuals from the Human Genome Diversity Project (HGDP).[15] Migrations of Middle Easterners and Central Asians into Europe were modelled as pulses occurring at varying time points, with rates ranging from 0 to 100%.

ABC estimation

We used an approximate Bayesian computation (ABC) approach[16,17] to estimate the age (T_{age}), the strength (s) and the onset (T_{onset}) of negative selection acting upon the *TYK2* P1104A mutation. Parameters were estimated using computer simulations best fitting the observed data. Simulated and empirical data were described by a vector of K summary statistics, which were used to fit the observed data. For each parameter, posterior

distributions, point estimates (in this case, posterior mode) and the 95% CIs were obtained from accepted simulated parameter values, that is, parameter values obtained from simulations where summary statistics are in close match with those of the empirical data. We used the 'abc' R package and the standard ABC method,[18,19] for which the accepted simulated parameters were subsequently adjusted by local linear regression (method = "Loclinear" in the 'abc' package). In this study, summary statistics included allele frequencies from simulated frequency trajectories. We modified the R package to allow matches between simulated and empirical summary statistics by means of weighted (based on sample sizes by epochs) Euclidean distances. Parameter estimations were obtained from 10,000,000 simulations drawn with predefined prior distributions for each parameter (priors are specified in the sections 'Age and origin estimation for the *TYK2* P1104A mutation' and 'Onset and strength of negative selection estimation for *TYK2* P1104A'). Accepted parameters were those of simulations providing the 0.01% lowest Euclidean distances between observed and simulated statistics (the 'abc' parameter 'tol' was set to be equal to 0.0001). Thus, ABC point estimates and 95% CIs for the age of the mutation, the strength and onset of selection (see below) were computed from the 1,000 best fitting simulations (0.01% of the total 10,000,000).

Age and origin estimation for the *TYK2* P1104A mutation

Age estimation (T_{age}) was obtained by ABC, using simulated frequency trajectories with or without selection (see section 'Onset and strength of negative selection estimation for *TYK2* P1104A'). As summary statistics, we considered all $K = 12$ available frequency estimations from the frequency trajectory, including ancient (from Paleolithic to Middle Ages) and modern DNA data (from Sub-Saharan Africa, Europe, Middle-East, Central-Asia and East-Asia) (**Table S3**). We removed archaic individuals (N=4, aged >40,000 ya) to the estimation of T_{age}, and assumed a uniform prior distribution for the age of the mutation over the last 100,000 year and a single origin for the *TYK2* P1104A mutation. Because the first occurrence of the allele in our dataset was observed 8,500 ya, we randomly sampled T_{age} from $\mathcal{U}[8.5\text{-}100]$ kya.

Onset and strength of negative selection estimation for *TYK2* P1104A

We assumed a relative fitness for each *TYK2* genotype of $w_{WT/WT} = 1$, $w_{WT/P1104A} = 1$, and $w_{P1104A/P1104A} = 1 - s$, consistent with experimental and epidemiological data suggesting a recessive effect of P1104A on disease risk.[20,21] In the analysis of selection (onset and

strength), we considered three simulation sets (**Table S4**). Consistent with previous dating of *M. tuberculosis* during the Neolithic,[22,23] we first explored the hypothesis of an early onset of negative selection. We performed 1,000,000 simulations assuming a continuous and constant negative selection on homozygous carriers starting 10,000 ya, by randomly sampling *s* from $\mathcal{U}[0\text{-}1]$ and fixing T_{onset} to 10,000 ya ("Early onset" set; **Table S4**). We then compared the early onset set with a second simulation set with more recent onsets of negative selection (varying from 10,000 ya to 500 ya, 250 years being the generation unit in our rescaled model). We performed 10,000,000 simulations randomly drawing *s* and T_{onset} from the uniform distributions $\mathcal{U}[0\text{-}1]$ and $\mathcal{U}[500\text{-}10{,}000]$, respectively ("Full" simulation set, Table S4). For this set, we performed ten times more simulations in order to better explore the parameter space, which is much larger in this case since T_{onset} also varies across simulations. In both cases, the allele was assumed to be neutral before the onset of selection. Here, a two-sampled Hotelling's T-squared test was used to assess whether the simulations best fitting the empirical data were drawn from the same distribution than that of *TYK2* P1104A (considering P1104A's trajectory as curves within its uncertainty frequency interval, **Table S3**). Finally, a third simulation set was conducted assuming a neutral evolution of the *TYK2* P1104A allele, by performing 1,000,000 simulations fixing *s* to 0 ("Neutral" simulation set, **Table S4**). This simulation set was compared to the "Full" set to assess the likelihood of negative selection in the evolution of the P1104A allele (see section 'Odds ratio (OR) computation').

The ABC estimations of *s* and T_{onset} were performed using the aforementioned "Full" set of 10,000,000 simulations and European summary statistics from the Late Neolithic epoch onwards (K=5; **Table S3**), as described in the main text. To account for population stratification, we considered a north-south geographical division for Europe. Specifically, we defined "northern Europe" to be the region east of 13°E and north of 45°N, or west of 13°E and north of 49°N, with "southern Europe" as the complement, as previously proposed.[11]

Because of the adopted rescaled model (see section 'Simulated *TYK2* P1104A frequency trajectory'), we used rescaled λs. Specifically, we used an iterative algorithm to obtain selection coefficients for the rescaled model, designed to provide expected trajectories of the selected allele under a non-rescaled model. Briefly, parameters of the model were multiplied by a factor λ (here $\lambda = 10$) to account for the rescaling process. However, if $s > 0.1$, $\lambda s > 1$, which lies outside the accepted range of values for selection coefficients ([0-1]). To compensate for this, we modified the dominance coefficient λh of the rescaled model for values of $s > 0.1$. We first observed the relationship between frequency changes from one

generation t to the next (t+1) for a biallelic locus with alleles 'a' and 'A'. We assumed a relative fitness for each genotype of $P(r_t|AA)$ = 1, $P(r_t|aA)$= 1 - hs, and $P(r_t|aa)$ = 1 − s.

$$P_{t+1} = P(aa|r_t) + \frac{1}{2}P(aA|r_t),$$

where frequency of allele a at time t is denoted P_t (and that of time t+1, P_{t+1}), and r_t is the Boolean event of either contributing or not to the offspring pool at generation t+1. Also,

$$P(aa|r_t) = \frac{P(r_t|aa)P(aa)}{P(r_t|aa)P(aa) + P(r_t|aA)P(aA) + P(r_t|AA)P(AA)}$$
$$= \frac{(1-s)P_t^2}{(1-s)P_t^2 + 2(1-hs)P_t(1-P_t) + (1-P_t)^2}.$$

Repeating this process for the heterozygous genotype and adding accordingly, we obtain,

$$P_{t+1} = \frac{(1-s)\,P_t^2 + (1-hs)(1-P_t)}{(1-s)\,P_t^2 + 2(1-hs)P_t(1-P_t) + (1-P_t)^2}. \quad (1)$$

Now, simply using formula (1) we estimate λh_t, which is the dominance coefficient at generation t in the rescaled model, to accommodate for the true frequency at generation t, denoted P_t, assuming s >0.1. To do so, if t0 is the first generation in the rescaled model at which s >0.1, we repeat iteratively λ-1 times formula (1) starting with $P'_{t0} = P_{t0\times\lambda}$, s and h = 0, to obtain the expected frequency at generation t0+1, denoted P'_{t0+1}. Using known P'_{t0} and $\lambda s = -1$, we derive the value of λh_{t0} as follows,

$$\lambda h_{t0} = 1 - \frac{P_{t0+\lambda-1}(1-P'_{t0})^2}{(1-2P_{t0+\lambda-1})P_{t0}(1-P'_{t0})}.$$

Analogously, we obtained λh_t for all generations t for which s >0.1.

Odds ratio (OR) computation

To localize the origin of the P1104A mutation, we focused on the "Full" simulation set (**Table S4**), from which we obtained estimates for T_{age}. We compared the proportion of

variants arising in the common ancestors of West Eurasians (WE) in the 1,000 best fitting simulations with that of the remaining 99,999,000 simulations. Then, we computed an OR from the following 2 x 2 contingency table:

Simulations \ Origin	Common Ancestor WE	Otherwise
**Best Fitting**	606	394
**Others**	1,806,191	8,192,809

We also provided evidence in favor of a negatively selected evolution for P1104A by comparing the "Full" with the "Neutral" simulation set (**Table S4**). We estimated, for each set, the proportion of best fitting simulations that had a frequency decrease at least as much as that of P1104A since the Bronze Age. We then computed an OR from the following 2 x 2 contingency table:

Simulations \ Frequency decrease	At least as P1104A	Otherwise
**Best Fitting Full**	250	750
**Best Fitting Neutral**	1	99

The ORs, the 95% CI and the chi square *p*-value were computed using the R (version 3.6.0) package "epitools". The corresponding OR was computed as follow

OR $= \frac{250/(1000-250)}{1/(100-1)} = 33$ (95% CI = [5-240], $p < 10^{-10}$).

Historical burden of TB in Europe due to homozygosity for *TYK2* P1104A

Using our point estimations for the onset and the strength of negative selection on *TYK2* P1104A, we sought to assess the historical number of *TYK2* P1104A-related deaths. This number was assessed following a number of assumptions: 25,000,000 individuals in Europe 2,000 ya,[24] a frequency of 0.05 for *TYK2* P1104A 2,000 ya, consistent with our aDNA data, and a previously defined exponential growth coefficient for Europeans,[6] leading to a realistic 520,000,000 Europeans today. The number of homozygotes at each generation (25 years) was obtained using Hardy-Weinberg equilibrium and the respective European sample size ($f^2\times$ sample size), the allele being in Hardy-Weinberg equilibrium in the current generation. We

also assumed that the relative 20% loss of fitness among homozygotes due to TB was either 100% or 50% lethal, so that 20% or 10% of *TYK2* P1104A homozygotes died at each generation due to TB. We further assessed the proportion of TB-related deaths due to *TYK2* P1104A assuming a constant mortality rate of TB of 800 in 100,000 until 200 ya.[25]

Factor Analysis

Factor analysis[26] was conducted on a merged data set consisting of 143,081 SNP genotypes for 363 present-day European individuals consisting mainly on the 1,000 Genomes Project individuals (IBS, TSI, GBR and FIN, available at the V42.4: March 1 2020 release) and 701 ancient samples from the aDNA dataset (see section 'Ancient DNA and present-day samples'). Factors are interpreted as principal components from principal component analysis but with temporal correction for present-day and ancient samples. The samples had ages less than 14,000 years old, and were chosen for their higher level of genomic coverage (>0.9X), as previously proposed.[26] Following this approach, we chose the drift parameter in order to remove the effect of time on the Kth factor (K = 4 here), where K is the number of ancestral groups considered.

Accounting for ancestry in the ABC estimation

We re-estimated the selection coefficient (*s*) and the onset of selection (T_{onset}) using the previously defined ABC approach, accounting for the observed ancestry proportions across epochs. To do so, we estimated ancestry proportions for ancient and modern samples by epoch, based on the admixture rates obtained from the previously described Factor Analysis,[26] using the 'ancestry_coefficients' function of the 'tfa' R (version 3.6.0) package. We used as European source populations either 21 Mesolithic hunter-gatherers (group ID 'Serbia_Mesolithic_IronGates') and 18 Anatolian (group ID 'Anatolia_N') farmers (until 5,000 ya) or the 18 Anatolian farmers and 1 herder (group ID 'Russia_Caucasus_EBA_Yamnaya') from the Steppes (after 5,000 ya) (**Table S1**), the proportion of Mesolithic hunter-gatherer ancestry at the population level being comparatively very low after the Bronze Age.[10]. We then summarized the estimations obtained for the Anatolian ancestry component as a frequency trajectory over time (**Table S3**).

Aside from the previously used summary statistics, we included to the ABC approach the average Anatolian ancestry proportions estimated at the Late Neolithic, the Bronze Age, the Iron Age, the Middle Ages and present times. To derive a simulated Anatolian ancestry trajectory of the sample, we added to the source Anatolian (MDE, **Figure 2B**) simulated

population a fixed marker variant that uniquely identifies individuals from this population, and that we linked to the variant under study (complete linkage disequilibrium between the simulated selected variant and the ancestry marker). We then used the frequency of the aforementioned marker variant in the EUR (**Figure 2B**) population to provide an estimation of the Anatolian ancestry proportion of the sample by epoch in Europe.

Reference bias in aDNA

The estimation of the allele frequency trajectory relies on the quality of the reads obtained from aDNA. In short-read mapping and in the process of in-solution enrichment, there may be a bias towards one of the two alleles present at a polymorphic site,[27] which usually favors the reference allele (the ancestral allele for the *TYK2* P1104 position). The combination of modern (without this issue) and aDNA data may therefore result in selection estimation bias, which, in the case of a bias favoring the reference allele, would result in an underestimation of s in our approach. We therefore removed modern data from our analysis and re-estimated s for northern, southern or all Europeans together.

Supplemental References

1. 1000 Genomes Project Consortium (2015). A global reference for human genetic variation. Nature *526*, 68-74.
2. Scott, E.M., Halees, A., Itan, Y., Spencer, E.G., He, Y., Azab, M.A., Gabriel, S.B., Belkadi, A., Boisson, B., Abel, L., et al. (2016). Characterization of Greater Middle Eastern genetic variation for enhanced disease gene discovery. Nat Genet *48*, 1071-1076.
3. Marchi, N., Mennecier, P., Georges, M., Lafosse, S., Hegay, T., Dorzhu, C., Chichlo, B., Segurel, L., and Heyer, E. (2018). Close inbreeding and low genetic diversity in Inner Asian human populations despite geographical exogamy. Sci Rep *8*, 9397.
4. Haller, B.C., and Messer, P.W. (2019). SLiM 3: Forward Genetic Simulations Beyond the Wright–Fisher Model. Mol Biol Evol *36*, 632-637.
5. Hoggart, C.J., Chadeau-Hyam, M., Clark, T.G., Lampariello, R., Whittaker, J.C., Iorio, M.D., and Balding, D.J. (2007). Sequence-Level Population Simulations Over Large Genomic Regions. Genetics *177*, 1725-1731.
6. Gravel, S., Henn, B.M., Gutenkunst, R.N., Indap, A.R., Marth, G.T., Clark, A.G., Yu, F., Gibbs, R.A., Project, T.G., and Bustamante, C.D. (2011). Demographic history and rare allele sharing among human populations. Proc Natl Acad Sci U S A *108*, 11983-11988.
7. Skoglund, P., and Mathieson, I. (2018). Ancient Genomics of Modern Humans: The First Decade. Annu Rev Genomics Hum Genet *19*, 381-404.
8. Allentoft, M.E., Sikora, M., Sjögren, K.-G., Rasmussen, S., Rasmussen, M., Stenderup, J., Damgaard, P.B., Schroeder, H., Ahlström, T., Vinner, L., et al. (2015). Population genomics of Bronze Age Eurasia. Nature *522*, 167-172.
9. Kılınç, G.M., Omrak, A., Özer, F., Günther, T., Büyükkarakaya, A.M., Bıçakçı, E., Baird, D., Dönertaş, H.M., Ghalichi, A., Yaka, R., et al. (2016). The Demographic Development of the First Farmers in Anatolia. Curr Biol *26*, 2659-2666.
10. Mathieson, I., Alpaslan-Roodenberg, S., Posth, C., Szécsényi-Nagy, A., Rohland, N., Mallick, S., Olalde, I., Broomandkhoshbacht, N., Candilio, F., Cheronet, O., et al. (2018). The genomic history of southeastern Europe. Nature *555*, 197-203.
11. Mathieson, S., and Mathieson, I. (2018). FADS1 and the Timing of Human Adaptation to Agriculture. Mol Biol Evol *35*, 2957-2970.
12. Olalde, I., Brace, S., Allentoft, M.E., Armit, I., Kristiansen, K., Booth, T., Rohland, N., Mallick, S., Szécsényi-Nagy, A., Mittnik, A., et al. (2018). The Beaker phenomenon and the genomic transformation of northwest Europe. Nature *555*, 190-196.
13. Lazaridis, I., Patterson, N., Mittnik, A., Renaud, G., Mallick, S., Kirsanow, K., Sudmant, P.H., Schraiber, J.G., Castellano, S., Lipson, M., et al. (2014). Ancient human genomes suggest three ancestral populations for present-day Europeans. Nature *513*, 409-413.
14. Haak, W., Lazaridis, I., Patterson, N., Rohland, N., Mallick, S., Llamas, B., Brandt, G., Nordenfelt, S., Harney, E., Stewardson, K., et al. (2015). Massive migration from the steppe was a source for Indo-European languages in Europe. Nature *522*, 207-211.
15. Bergström, A., McCarthy, S.A., Hui, R., Almarri, M.A., Ayub, Q., Danecek, P., Chen, Y., Felkel, S., Hallast, P., Kamm, J., et al. (2020). Insights into human genetic variation and population history from 929 diverse genomes. Science *367*, eaay5012.
16. Beaumont, M.A. (2010). Approximate Bayesian Computation in Evolution and Ecology. Annu Rev Ecol Evol S *41*, 379-406.
17. Tavaré, S., Balding, D.J., Griffiths, R.C., and Donnelly, P. (1997). Inferring Coalescence Times From DNA Sequence Data. Genetics *145*, 505-518.
18. Beaumont, M.A., and Rannala, B. (2004). The Bayesian revolution in genetics. Nat Rev Genet *5*, 251-261.

19. Beaumont, M.A., Zhang, W., and Balding, D.J. (2002). Approximate Bayesian Computation in Population Genetics. Genetics *162*, 2025-2035.
20. Kerner, G., Ramirez-Alejo, N., Seeleuthner, Y., Yang, R., Ogishi, M., Cobat, A., Patin, E., Quintana-Murci, L., Boisson-Dupuis, S., Casanova, J.-L., et al. (2019). Homozygosity for TYK2 P1104A underlies tuberculosis in about 1% of patients in a cohort of European ancestry. Proc Natl Acad Sci U S A *116*, 10430-10434.
21. Boisson-Dupuis, S., Ramirez-Alejo, N., Li, Z., Patin, E., Rao, G., Kerner, G., Lim, C.K., Krementsov, D.N., Hernandez, N., Ma, C.S., et al. (2018). Tuberculosis and impaired IL-23–dependent IFN-γ immunity in humans homozygous for a common TYK2 missense variant. Sci Immunol *3*, eaau8714.
22. Bos, K.I., Harkins, K.M., Herbig, A., Coscolla, M., Weber, N., Comas, I., Forrest, S.A., Bryant, J.M., Harris, S.R., Schuenemann, V.J., et al. (2014). Pre-Columbian mycobacterial genomes reveal seals as a source of New World human tuberculosis. Nature *514*, 494-497.
23. Sabin, S., Herbig, A., Vagene, A.J., Ahlstrom, T., Bozovic, G., Arcini, C., Kuhnert, D., and Bos, K.I. (2020). A seventeenth-century Mycobacterium tuberculosis genome supports a Neolithic emergence of the Mycobacterium tuberculosis complex. Genome Biol *21*, 201.
24. Durand, J.D. (1974). Historical Estimates of World Population: An Evaluation.(University of Pennsylvania).
25. Dubos, R.J., and Dubos, J. (1987). The White Plague: Tuberculosis, Man, and Society.(Rutgers University Press).
26. Francois, O., and Jay, F. (2020). Factor analysis of ancient population genomic samples. Nat Commun *11*, 4661.
27. Gunther, T., and Nettelblad, C. (2019). The presence and impact of reference bias on population genomic studies of prehistoric human populations. PLoS Genet *15*, e1008302.
