# Supplementary material for: Human ancient DNA analyses reveal the high burden of tuberculosis in Europeans over the last 2,000 years
Source: Am J Hum Genet. 2021 Mar 4;108(3):517–24. doi: 10.1016/j.ajhg.2021.02.009 (PMC8008489; doi:10.1016/j.ajhg.2021.02.009)
Supplement: Document S2. Article plus supplemental information [file mmc3.pdf]

REPORT

Human ancient DNA analyses reveal the high burden of tuberculosis in Europeans over the last 2,000 years

Gaspard Kerner,[1,2,3,*] Guillaume Laval,[1] Etienne Patin,[1] Stéphanie Boisson-Dupuis,[2,3,4] Laurent Abel,[2,3,4] Jean-Laurent Casanova,[2,3,4,5,7] and Lluis Quintana-Murci[1,6,7,*]

Summary

Tuberculosis (TB), usually caused by *Mycobacterium tuberculosis* bacteria, is the first cause of death from an infectious disease at the worldwide scale, yet the mode and tempo of TB pressure on humans remain unknown. The recent discovery that homozygotes for the P1104A polymorphism of *TYK2* are at higher risk to develop clinical forms of TB provided the first evidence of a common, monogenic predisposition to TB, offering a unique opportunity to inform on human co-evolution with a deadly pathogen. Here, we investigate the history of human exposure to TB by determining the evolutionary trajectory of the *TYK2* P1104A variant in Europe, where TB is considered to be the deadliest documented infectious disease. Leveraging a large dataset of 1,013 ancient human genomes and using an approximate Bayesian computation approach, we find that the P1104A variant originated in the common ancestors of West Eurasians ~30,000 years ago. Furthermore, we show that, following large-scale population movements of Anatolian Neolithic farmers and Eurasian steppe herders into Europe, P1104A has markedly fluctuated in frequency over the last 10,000 years of European history, with a dramatic decrease in frequency after the Bronze Age. Our analyses indicate that such a frequency drop is attributable to strong negative selection starting ~2,000 years ago, with a relative fitness reduction on homozygotes of 20%, among the highest in the human genome. Together, our results provide genetic evidence that TB has imposed a heavy burden on European health over the last two millennia.

Infectious diseases have been the leading cause of mortality since the origin of modern humans in Africa and throughout their subsequent dispersals around the world.[1–5] Tuberculosis (TB [MIM: 607948]) is considered to be the deadliest infection of the common era, with more than one billion deaths over the last 2,000 years,[6–8] and still responsible for more than 1.5 million deaths annually according to the WHO. The human genetic basis of TB susceptibility has remained elusive until the turn of the 21st century, when two rare inborn errors of immunity, autosomal-recessive interleukin-12 receptor b1 (IL-12Rb1) and tyrosine kinase 2 (TYK2) deficiencies, were identified in children with severe TB.[9,10] It was only in 2018 that the first common, monogenic predisposition to TB was identified. Homozygotes for the *TYK2* (MIM: 611521) P1104A polymorphism (rs34536443) were found to be at higher risk of developing clinical forms of TB, due to the selective disruption of IL-23-dependent antimycobacterial IFN-g immunity, underlying a recessive trait.[11] A subsequent study revealed an enrichment in P1104A homozygotes among TB cases of a case-control cohort from the United Kingdom, where the allele is most prevalent today (4%).[7] The frequency of P1104A, together with its high penetrance for TB in the homozygous state (>0.8),[11] suggests that about 1/600 British individuals would develop TB during their lifetime because of the mutation, if TB were still highly endemic in Europe.

Pathogen-imposed selective pressures have been paramount during human evolution.[2,4,5] Over the last decade, population genetic studies have documented strong, distinct selection signatures among host defense genes, helping to delineate immunological mechanisms of major importance,[12] and supporting the notion that microbes have had an overwhelming impact on human genome diversity.[4,5] While several studies have provided insight into the periods when malaria has exerted pressure on humans,[13–17] little is known about the historical burden of other infectious diseases associated with past epidemics. Yet, TB appears to have been more lethal than malaria in the common era,[6] making it a stronger selective pressure in endemic regions. Recent evidence based on mycobacterial ancient DNA (aDNA) suggests a Holocene dispersal of *M. tuberculosis* <6,000 years ago (ya),[18,19] a time frame that coincides with the growth of agricultural communities and anthropogenic environmental changes, which may have favored infectious disease transmission.[20]

To investigate the historical burden of TB in humans, we sought to reconstruct the evolutionary history of the *TYK2* P1104A variant. Indeed, this mutation, in the homozygous state, underlies the only known common, monogenic predisposition to TB.[7,11] Moreover, *TYK2* P1104A does not affect the risk for other infectious diseases except, to a milder degree, rare cases of infection by environmental mycobacteria in otherwise healthy individuals.[11] Whereas

[1]Human Evolutionary Genetics Unit, Institut Pasteur, UMR2000, CNRS, 75015 Paris, France; [2]Laboratory of Human Genetics of Infectious Diseases, Necker Branch, INSERM UMR 1163, Necker Hospital for Sick Children, 75015 Paris, France; [3]Paris University, Imagine Institute, 75015 Paris, France; [4]St. Giles Laboratory of Human Genetics of Infectious Diseases, Rockefeller Branch, Rockefeller University, New York, NY 10065, USA; [5]Howard Hughes Medical Institute, New York, NY 10065, USA; [6]Chair of Human Genomics and Evolution, Collège de France, 75005 Paris, France
[7]These authors contributed equally
*Correspondence: gakerner@pasteur.fr (G.K.), quintana@pasteur.fr (L.Q.-M.)
https://doi.org/10.1016/j.ajhg.2021.02.009.
© 2021 The Author(s). This is an open access article under the CC BY license (http://creativecommons.org/licenses/by/4.0/).

disease-protective variants may rapidly increase in frequency owing to positive Darwinian selection, disease-risk alleles are expected to evolve under strong negative selection and be gradually purged from the population. Because negatively selected variants have become rare, very rare, or even extinct, they are harder to study using genetic data from modern human populations. However, with the increasing availability of genomes from ancient individuals, direct measurements of the intensity of selection are now possible, as significant increases or decreases of allele frequencies can be captured with aDNA from time transects.[21] Thus, the study of the P1104A variant offers an unprecedented opportunity to shed light on the evolutionary history of a deadly human disease such as TB. Of note, P1104A homozygotes have also been shown to enjoy from a protective effect against various autoimmune and inflammatory diseases.[22,23] While this effect could have provided a fitness advantage opposed to that attributable to TB infection, the general late onset manifestation of autoimmune and inflammatory disorders makes unlikely the occurrence of a large counteractive effect.

We therefore examined the frequency trajectory of P1104A over the last 10,000 years of European history, by screening a collection of 1,013 genomes that cover a time transect from the Mesolithic period to the Middle Ages (Figure 1A; Table S1). We partitioned the aDNA data into seven epochs and incorporated data from present-day populations (supplemental material and methods). The P1104A variant, which we found to be the result of a single mutational event (Figure S1), appeared for the first time in our dataset during the early Neolithic ~8,500 ya in the Anatolian peninsula, and then spread to Central Europe where it remained at frequencies lower than 3% until ~5,000 ya (Figures 1A–1C). During the Bronze Age, P1104A increased in frequency, reaching its maximum frequency ~3,000 ya at nearly 10%. After the Iron Age, we observed a strong and consistent decrease in frequency of P1104A, resulting in an average frequency of 2.9% among contemporary Europeans.[24]

We estimated the age of the *TYK2* P1104A mutation (T_{age}), tested whether the mutation has been the substrate of natural selection, and inferred the onset (T_{onset}) and strength (s) of negative selection acting on homozygotes, using an approximate Bayesian computation (ABC) approach[25] that considers large prior assumptions (T_{age} $\sim\mathcal{U}$[8.5–100,000] ya, T_{onset} $\sim\mathcal{U}$[500–10,000] ya and s $\sim\mathcal{U}$[0–1]; supplemental material and methods). We first determined the extent to which our approach could determine the evolutionary model of P1104A that best explains the observed aDNA data, by comparing the fit of the simulated to the observed data (supplemental material and methods). We assumed a validated demographic model for Europeans,[26] to which we added gene flow from both Near Easterners and Central Asians (Table S2), to account for the large-scale migrations of early farmer populations of the Anatolian plateau and Eurasian steppe populations associated with the Yamnaya culture inferred from aDNA.[27] In doing so, considering the aforementioned large prior assumptions, we obtained simulated frequency trajectories that closely reproduce that of P1104A, similarly to other genome-wide variants (Figure S2). We also noted a similar, or higher, increase in frequency as that observed for P1104A until the Bronze Age for more than 20% of other aDNA variants within the uncertainty frequency interval of P1104A in the Mesolithic ([0.00–0.10]; Table S3), highlighting the marked impact of the aforementioned migratory events on the frequency of a large fraction of genomic variants, including P1104A. Furthermore, simulated neutral variants closely matched observed frequency distributions of non-coding variants for all epochs (Figure S3), indicating that the demographic model used—present-day Europeans are a mixture of Mesolithic hunter-gatherers, Anatolian Neolithic farmers, and Eastern steppe-related groups[28,29]—well reproduces the neutral patterns of European diversity.

We then estimated the origin of the *TYK2* P1104A mutation, based on its frequency in $K = 12$ populations sampled at different epochs, including European aDNA data (Paleolithic, Mesolithic, Early Neolithic, Late Neolithic, Bronze Age, Iron Age, and Middle Ages; supplemental material and methods) and present-day Europeans, Middle Easterners, Central Asians (from 1% to 4%), Sub-Saharan Africans (0%), and East Asians (0%) (Figure 2A; Table S3). We found the age of P1104A to be ~30,000 years old (mode = 29,182; 95% CI [20,636–57,285]) (Figure 2B; supplemental material and methods), which is consistent with a previous estimate.[30] Using cross-validation, we found that parameter estimation was accurate across all ages, with 96% of 1,000 estimated 95% CIs including the true simulated value, and also robust to the choice of the summary statistics used (Figures S4A–S4D). While the 95% CI for the age of P1104A overlaps with the divergence time between West and East Eurasians (35–45 kya), the proportion of best-fitting simulated variants originating in the common ancestors of West Eurasians was significantly higher than that of the rest of simulated variants (OR = 7.00, 95% CI [5.70–8.53], $p < 10^{-10}$; Figure 2B; supplemental material and methods). This suggests that P1104A originated in the common ancestors of West Eurasians after the split with East Eurasians, but before the divergence of Europeans, Middle Easterners, and Central Asians. Together, our results provide robust evidence that *TYK2* P1104A appeared during the Upper Paleolithic in West Eurasia, largely predating the estimated emergence of TB in Europe.[18,19,31]

We next investigated the evolutionary forces that could explain the frequency decrease of P1104A since the Bronze Age, where the maximum frequency is observed, by simulating frequency trajectories under neutrality ($s = 0$) or negative selection ($s > 0$) (Figure S5A). We found that simulations matching the estimated frequency of P1104A at the end of the Bronze Age explained both the observed aDNA and modern data only if $s > 0.1$. Furthermore, the frequency decrease after the Bronze Age was observed in

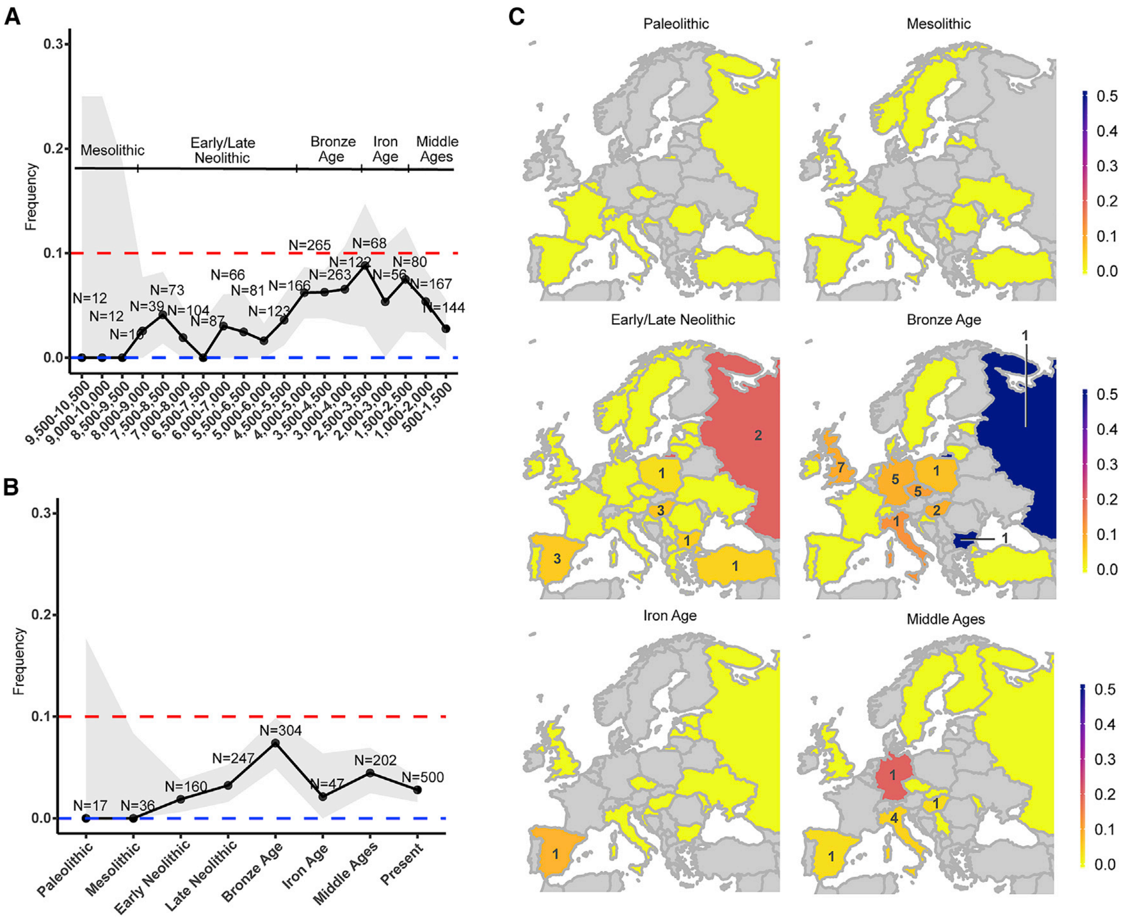

Figure 1. Evolutionary history of the TB-associated *TYK2* P1104A variant

(A and B) European frequency trajectory for the *TYK2* P1104A variant over the last 10,000 years for (A) bins of 1,000 years and sliding windows of 500 years or (B) seven (pre-) historical European epochs and current times. The red and blue horizontal dashed lines indicate a frequency of 10% and 0%, respectively. Uncertainty of the frequency estimation is indicated by a gray colored area, representing the normal approximation of the 95% binomial proportion CI. Large uncertainty for older times is due to small sample sizes. For each bin, at least one carrier was assumed to obtain uncertainty estimates.

(C) Geographical distribution of the *TYK2* P1104A allele by country (using today's political borders), across all defined epochs. Colors indicate frequency estimations by country, from 0 (yellow) to 0.5 (blue). Grey indicates unavailable data. Number of P1104A carriers is indicated with its respective number on each country. Sample sizes for countries with non-zero counts (Table S1) are the following: Early/Late Neolithic: Austria (n = 7), Bulgaria (n = 21), Croatia (n = 10), Czech Republic (n = 8), Denmark (n = 1), Estonia (n = 1), France (n = 4), Germany (n = 13), Greece (n = 9), Hungary (n = 51), Ireland (n = 4), Italy (n = 11), Latvia (n = 20), Lithuania (n = 8), Luxembourg (n = 1), Macedonia (n = 1), Norway (n = 1), Poland (n = 32), Portugal (n = 11), Romania (n = 3), Russia (n = 10), Serbia (n = 14), Spain (n = 57), Sweden (n = 11), Turkey (n = 22), UK (n = 46), Ukraine (n = 27); Bronze Age: Bulgaria (n = 2), Croatia (n = 2), Czech Republic (n = 46), Denmark (n = 2), Estonia (n = 7), France (n = 6), Germany (n = 58), Hungary (n = 17), Ireland (n = 1), Italy (n = 8), Lithuania (n = 4), the Netherlands (n = 10), Poland (n = 15), Portugal (n = 2), Russia (n = 2), Spain (n = 33), Sweden (n = 7), Switzerland (n = 1), Turkey (n = 5), UK (n = 75); Iron Age: Bulgaria (n = 1), Croatia (n = 1), Czech Republic (n = 1), Estonia (n = 3), Hungary (n = 5), Italy (n = 6), Latvia (n = 8), Moldova (n = 4), Russia (n = 2), Spain (n = 12), UK (n = 1); Middle Ages: Czech Republic (n = 1), Finland (n = 4), Germany (n = 5), Hungary (n = 30), Iceland (n = 9), Italy (n = 89), Moldova (n = 2), Russia (n = 3), Serbia (n = 1), Slovakia (n = 1), Spain (n = 32), Sweden (n = 13), UK (n = 12).

the trajectories of 25% of the best fitting simulated deleterious variants ($s \sim \mathcal{U}[0\text{–}1]$ and $T_{onset} \sim \mathcal{U}[500\text{–}10{,}000]$; supplemental material and methods), relative to only 1% of the best fitting simulated neutral variants (OR = 33, 95% CI = [5–240], $p < 10^{-10}$; Figure S5B; Table S4). These observations collectively support a history of negative selection driving the evolution of the TB-risk P1104A variant after the Bronze Age.

To quantify the degree of deleteriousness of *TYK2* P1104A during European history, we verified that allele frequency trajectories were informative to assess negative selection, and, encouragingly, we observed a strong positive correlation between drops in allele frequencies and *s* values (Figure S6A). We first hypothesized that negative selection started with the arrival of agriculture in Europe,[20] a period that includes the upper bound estimation for the most recent common ancestor of the *M. tuberculosis* complex ~6,000 ya.[18,19] However, such an early onset of selection ($T_{onset} = 10{,}000$) was clearly rejected by our simulations (Hotelling's T-squared test $p = 5.4 \times 10^{-4}$; Figure S6B; supplemental material and methods; Table S4), as no simulated variants were able to reproduce the frequency increase of P1104A until the Bronze

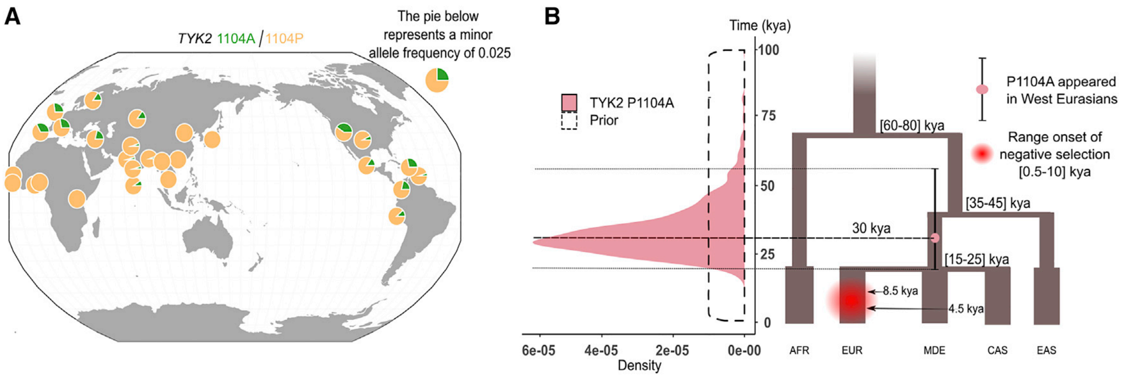

Figure 2. Present-day geographic distribution and age estimation of the *TYK2* P1104A mutation

(A) Frequencies from present-day Europeans (EUR; f = 0.03; n = 503), sub-Saharan Africans (AFR; f = 0; n = 504), East Asians (EAS; f = 0; n = 504), Americans (AMR; f = 0.01; n = 347), Middle Easterners (MDE; f = 0.02; n = 163), and Central Asians (CAS; f = 0.01; n = 363) are shown (supplemental material and methods). Figure was built with 1000 Genomes Project data[24] and modified to include Middle Easterners and Central Asians. The presence of *TYK2* P1104A among American populations from the 1000 Genomes Project reflects recent admixture with Europeans,[24] with all populations sharing a unique 6 kb-long haplotype around *TYK2* P1104A, although the allele is absent from Native Americans.

(B) Left panel: posterior distribution for the age (in thousands of years) of the *TYK2* P1104A mutation, according to the best fitting simulations with variable onset of selection, using 10,000,000 simulations and all available summary statistics. CI boundaries are shown with dashed black lines. Right panel: the proposed demographic model, showing the point estimate for T_{age} (mode = 30 kya, purple red circle) and the 95% confidence interval (black vertical segment across the purple circle) for the age of the mutation.

Age. Conversely, when allowing the onset of selection to vary across the last 10,000 years, using the former large priors ($T_{onset} \sim \mathcal{U}[500–10,000]$ ya and $s \sim \mathcal{U}[0–1]$), our best simulations did not significantly differ from P1104A (i.e., the simulation set was not rejected; Hotelling's T-squared test p = 0.09) and revealed that scenarios with recent onsets of negative selection were those best fitting the data (Figure S6B).

To explain the strongest frequency increase and decrease for P1104A, we modeled allele frequencies of K = 5 ancient populations (Late Neolithic, Bronze Age, Iron Age, and Middle Ages) and present-day Europeans, and assumed large priors for model parameters (supplemental material and methods, Table S3). We found that negative selection on P1104A homozygous carriers started 1,937 ya (95% CI [500–7,912]), with a selection coefficient of 0.21 (95% CI [0.06–0.82]) (Figures 3A–3C). This onset of selection is consistent with a neutral evolution for the allele until the Bronze Age, suggesting that drift and admixture are sufficient to explain the increase of P1104A frequency until this epoch. These estimations should not be biased owing to read mapping bias of the reference allele in the ancient genome dataset,[32] given that 1104A is the alternative allele (supplemental material and methods). Furthermore, parameter estimation was found to be robust to the choice of the summary statistics used, with the 95% CIs of the estimates including the true simulated value 93% of the time (Figures S6C and S6D). Although our analysis showed that the more recent the onset of selection was the closer the frequency trajectory estimation was to the empirical data (Figure S6A), the fit was found to be similar within the last ~2,000 years (Figure 3B), consistent with our estimation. With respect to the selection coefficient, the posterior distributions of s were shifted to 1 as T_{onset} became closer to 0, and the general posterior distribution for the strength of negative selection was similar to that of onsets of selection occurring between 1,000 and 3,000 ya (Figures 3A and 3C). Importantly, consistent ABC estimates of the strength and the onset of selection were found when either excluding the Iron Age, i.e., the epoch with smallest sample size (s = 0.19; 95% = [0.03–0.83]; T_{onset} = 1,670 ya; 95% CI = [500–8,388] ya) or when using the whole European frequency trajectory, i.e., from the Paleolithic to the present (s = 0.21; 95% = [0.04–0.84]; T_{onset} = 1,567 ya; 95% CI = [500–8,367]).

Using the same approach, we estimated the selection coefficient of another mutation, *TYK2* I684S, a missense variant that is neither in linkage disequilibrium with P1104A nor associated with TB risk,[11] and found values that were compatible with neutrality (s = 0.02; 95% CI [0–0.19]; Figures S7A and S7B). Thus, our analyses support the notion that, despite the reported protective effects of P1104A against some immune-related disorders,[22,23] TB has exerted pressure on the *TYK2* P1104A variant over the last ~2,000 years, with a 20% relative fitness reduction for homozygotes at each generation since.

Finally, we sought to apply the same approach to reported pathogenic variants, by cross-matching the ClinVar database[33] with aDNA variants present in our cohort that fall into the uncertainty range of P1104A in the Bronze Age ([0.04–0.10], Figure 1B). Among the resulting three variants with a "pathogenic" clinical significance annotation, only one (*HFE* C282Y [MIM: 613609]) presents a frequency decrease across the last four epochs. *HFE* C282Y is a known disease-causing variant underlying hemochromatosis, an autosomal-recessive autoimmune disease (HFE1 [MIM: 235200]) that impairs mineral metabolism, which can affect the growth and clearance of intra- and extra-cellular pathogens.[34] *HFE* C282Y reached its maximum frequency, of

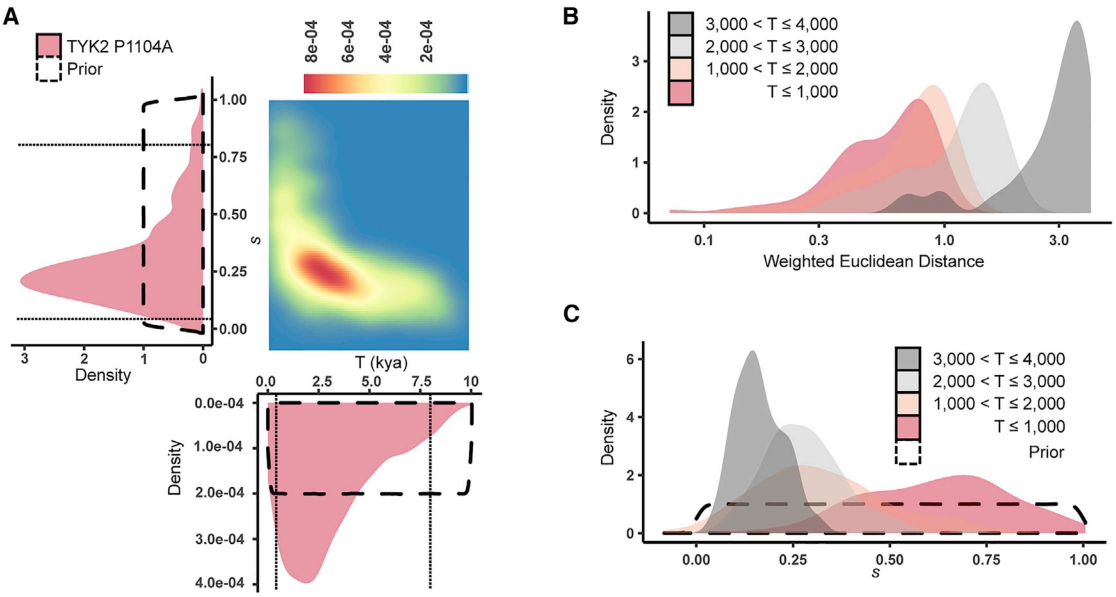

Figure 3. Estimation of the onset and strength of negative selection driving the evolution of *TYK2* P1104A
(A) Joint (as a 2D density plot) and individual (as purple densities) posterior distributions for the onset (in thousands of years) and the strength of negative selection (*s*) for *TYK2* P1104A, based on the best fitting simulations with variable onset of selection, using European summary statistics from the Late Neolithic epoch onward (10,000,000 simulations). CI boundaries are shown with dashed black lines.
(B) Distribution of the weighted Euclidean distances between the best fitting simulations and the observed data, under the proposed demographic model, for (from right to left) $3{,}000 < T_{onset} \leq 4{,}000$, $2{,}000 < T_{onset} \leq 3{,}000$, $1{,}000 < T_{onset} \leq 2{,}000$, or $500 < T_{onset} \leq 1{,}000$.
(C) Posterior distributions for the *TYK2* P1104A's negative selection coefficient, based on the best fitting simulations with variable onset of selection, for the same groups of onsets of selection as in (B) using the same color code.

nearly 10%, during the Middle Ages and then decreased to its present-day frequency of 4%. Consistent with our expectations, we found a similarly strong selection coefficient of 0.20 (mode = 0.22; 95% CI [0.03–0.76]; Figure S7B), and an onset of negative selection during the Middle Ages (mode = 724 ya; 95% CI [500–7,508]).

A potential limitation of our approach, which is inherent to most aDNA studies, is genetic discontinuity due to large population replacements or to sampling bias for geographical locations.[35] For example, different sampling proportions from northern and southern Europeans across epochs may result in genetic discontinuity in our dataset, given that the former present higher Eastern steppe ancestry than the latter after the Bronze Age.[36] We thus repeated our ABC setup for northern and southern Europeans using a geographical division,[37] designed to distinguish high and low levels of Steppe ancestry (Figure S8). Despite much lower sample sizes, we found evidence for negative selection in both northern ($s = 0.24$; 95% CI: [0.02–0.87]) and southern ($s = 0.13$; 95% CI: [1.6×10^{-4}–0.81]) European homozygotes, with a slightly left-shifted posterior distribution in southern Europe, where the sample size is more limited (Figure S9). We also found, using factor analysis,[38] that P1104A carriers scattered throughout European sub-structured populations, across all epochs after its introduction to Europe (Figure 4).

In addition, ancestry proportions were similar between P1104A carriers and the rest of the dataset at each epoch (Table S1). Notably, the observed ancestry shift between Bronze Age and present-day samples (from 0.29 to 0.36 for the whole dataset [Table S3], representing a 24% relative increase, and from 0.23 to 0.39 for P1104A carriers [Table S1]) does not, on its own, explain the frequency decline of the allele after the Bronze Age (from 0.074 to 0.029, representing a 61% relative decrease). Yet, we performed an ABC estimation accounting for ancestry variation across epochs (supplemental material and methods). Using the estimated Anatolian ancestry of our dataset at each epoch from the Late Neolithic onward, we estimated very similar values for the strength and onset of negative selection for *TYK2* P1104A at the pan-European level ($s = 0.27$; 95% CI: [0.08–0.93]; $T_{onset} =$ 2,045 ya; 95% CI [500–8,690]; Figures S10A and S10B). Similarly, we found comparable estimations for northern and southern Europeans ($s = 0.26$; 95% CI [0.06–0.83]; $T_{onset} =$ 1,046 ya; 95% CI [500–6,934]; Figures S10C and S10D; and $s = 0.24$; 95% CI [0.02–0.85]; $T_{onset} =$ 3,229 ya; 95% CI [500–8,963]; Figures S10E and S10F, respectively). Conversely, we found no evidence of selection for *TYK2* I684S ($s = 0.02$; 95% CI: [0–0.69]), as expected, and a weaker signal of negative selection for *HFE* C282Y ($s = 0.12$; 95% CI: [0–0.76]). Collectively, these findings suggest that the observed frequency drop of P1104A after the Bronze Age is not due to major geographical and/or temporal differences in ancestry components in our aDNA dataset, but instead to the action of natural selection. Moreover, when re-estimating the age of P1104A without modern data from Middle Easterners and Central Asians, as they are not entirely representative of ancestral Anatolian farmers and steppe herders, respectively,[39,40] we obtained almost identical results (mode = 30,303 ya; 95% CI [23,113–60,273]).

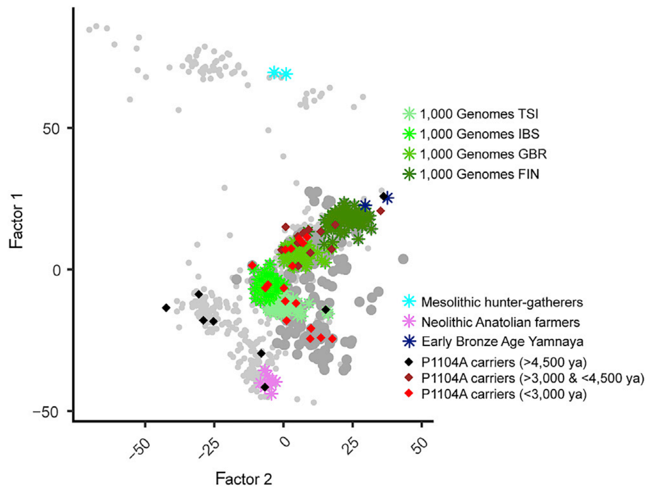

Figure 4. *TYK2* P1104A carriers scatter throughout the genetic diversity of the dataset
Factor analysis (Factor 2 versus Factor 1) for 701 high-quality ancient genomes from the full set of 1,013 used in this work, and 363 pseudo-haploid present-day genomes (TSI, IBS, GBR, and FIN, green colors from lighter to darker, respectively) from various European populations from the 1000 Genomes Project (supplemental material and methods). Two Mesolithic hunter-gatherers (cyan), eight Neolithic Anatolian farmers (violet), and two Early Bronze Age individuals associated with the Yamnaya (>80% steppe ancestry) culture (blue) account for the three major ancestries existing in present-day Europeans, which are, in turn, correlated with their respective epochs. P1104A carriers are shown with black (>4,500 ya), brown (>3,000 ya and <4,500 ya), or red (<3,000 ya) diamonds. Other individuals, older (light) or younger (dark) than 3,000 ya, are represented by gray dots.

In this attempt to define the mode and tempo of TB pressure on Europeans, we found that the only common variant known to underlie monogenic predisposition to TB has evolved under strong negative selection in Europe after the Iron Age. In doing so, we provide population genetic evidence for the high burden of TB in Europeans over the last two millennia, in line with the dating of *M. tuberculosis* lineage 4 at 1,943 ya[41] and of strains found in 18th century Hungarian mummies at 1,604 ya, or in mummified remains of the 17th century Bishop Peder Winstrup of Lund between 929 and 2,084 ya.[19,31] Notably, the TB-associated mutation ranks among the top 2.7% of variants, present in the studied capture array, with similar frequencies in the Bronze Age (0.04–0.10) that have decreased the most since this period (Table S3; supplemental material and methods). Such variants might also include targets of negative selection (Table S5). A selection coefficient of 0.20 would entail >2,500,000 cumulative deaths over the last 2,000 years due to P1104A homozygosity, representing 1%–2% of all TB-related deaths in the 19th century Europe (Figure S11). This figure is consistent with a previous estimation of 1% of TB cases due to the at-risk genotype among present-day Europeans.[7] We anticipate that the same population genetics framework could be used to delineate other human genetic variants, of yet unknown function, that have drastically decreased or increased in frequency across time due to microbial pressure. Thus, adopting an evolutionary approach represents a promising alternative to investigate the genetic sources of present-day disparities, between individuals and populations, in susceptibility to infection.

Data and code availability

Pseudo-haploid ancient and modern genome data are available at https://reich.hms.harvard.edu/allen-ancient-dna-resource-aadr-downloadable-genotypes-present-day-and-ancient-dna-data (V42.4: March 1, 2020 release). Code to perform ABC estimations from simulated frequency data are available at https://github.com/h-e-g/SLiM_aDNA_selection.

Supplemental Information

Supplemental Data can be found online at https://doi.org/10.1016/j.ajhg.2021.02.009.

Acknowledgments

We thank David Reich for helpful comments on an earlier version of the manuscript. We also thank Guillaume Achaz, Jérémy Choin, Evelyne Heyer, Nina Marchi, Anna-Lena Neehus, Olivier Neyrolles, and Mary O'Neill for data sharing and discussions. The Laboratory of Human Genetics of Infectious Diseases is supported by the Howard Hughes Medical Institute, the Rockefeller University, the St. Giles Foundation, the National Institutes of Health (NIH) (R01AI088364), the Meyer Foundation, the Agence Nationale de la Recherche (ANR) under the "Investments for the Future" program (ANR-10-IAHU-01), the Fondation pour la Recherche Méd (FRM) (EQU201903007798), *Institut National de la Santé et de la Recherche Médicale* (INSERM), and the University of Paris. The laboratory of Human Evolutionary Genetics is supported by the Institut Pasteur, the Collège de France, the Centre Nationale de la Recherche Scientifique (CNRS), the Agence Nationale de la Recherche (ANR) grants LIFECHANGE (ANR 17 CE12 0018 02) and CNSVIRGEN (ANR-19-CE15-0009-02), the French Government's Investissement d'Avenir program, Laboratoires d'Excellence "Integrative Biology of Emerging Infectious Diseases" (ANR-10- LABX-62-IBEID) and "Milieu Intérieur" (ANR-10-LABX-69-01), the Fondation pour la Recherche Médicale (Equipe FRM DEQ20180339214), the Fondation Allianz-Institut de France, and the Fondation de France (n°00106080). G.K. was supported by the Imagine Institute with the grant "Imagine Thesis Award."

Declaration of Interests

The authors declare no competing interests.

Received: October 12, 2020
Accepted: February 5, 2021
Published: March 4, 2021

Web resources

OMIM, https://www.omim.org/

References

1. Anderson, R.M., May, R.M., and Anderson, B. (1992). Infectious Diseases of Humans: Dynamics and Control (Oxford: Oxford University Press).
2. Cairns, J., and Singer, A.L. (1997). Matters Of Life And Death: Perspectives On Public Health, Molecular Biology, Cancer, And The Prospects For The Human Race (Diane Pub Co).
3. Casanova, J.-L., and Abel, L. (2018). Human genetics of infectious diseases: Unique insights into immunological redundancy. Semin. Immunol. *36*, 1–12.
4. Karlsson, E.K., Kwiatkowski, D.P., and Sabeti, P.C. (2014). Natural selection and infectious disease in human populations. Nat. Rev. Genet. *15*, 379–393.
5. Quintana-Murci, L. (2019). Human Immunology through the Lens of Evolutionary Genetics. Cell *177*, 184–199.
6. Paulson, T. (2013). Epidemiology: A mortal foe. Nature *502*, S2–S3.
7. Kerner, G., Ramirez-Alejo, N., Seeleuthner, Y., Yang, R., Ogishi, M., Cobat, A., Patin, E., Quintana-Murci, L., Boisson-Dupuis, S., Casanova, J.-L., and Abel, L. (2019). Homozygosity for *TYK2* P1104A underlies tuberculosis in about 1% of patients in a cohort of European ancestry. Proc. Natl. Acad. Sci. USA *116*, 10430–10434.
8. Furin, J., Cox, H., and Pai, M. (2019). Tuberculosis. Lancet *393*, 1642–1656.
9. Boisson-Dupuis, S., Bustamante, J., El-Baghdadi, J., Camcioglu, Y., Parvaneh, N., El Azbaoui, S., Agader, A., Hassani, A., El Hafidi, N., Mrani, N.A., et al. (2015). Inherited and acquired immunodeficiencies underlying tuberculosis in childhood. Immunol. Rev. *264*, 103–120.
10. Abel, L., Fellay, J., Haas, D.W., Schurr, E., Srikrishna, G., Urbanowski, M., Chaturvedi, N., Srinivasan, S., Johnson, D.H., and Bishai, W.R. (2018). Genetics of human susceptibility to active and latent tuberculosis: present knowledge and future perspectives. Lancet Infect. Dis. *18*, e64–e75.
11. Boisson-Dupuis, S., Ramirez-Alejo, N., Li, Z., Patin, E., Rao, G., Kerner, G., Lim, C.K., Krementsov, D.N., Hernandez, N., Ma, C.S., et al. (2018). Tuberculosis and impaired IL-23-dependent IFN-γ immunity in humans homozygous for a common *TYK2* missense variant. Sci. Immunol. *3*, 3.
12. Quintana-Murci, L., and Clark, A.G. (2013). Population genetic tools for dissecting innate immunity in humans. Nat. Rev. Immunol. *13*, 280–293.
13. Kwiatkowski, D.P. (2005). How malaria has affected the human genome and what human genetics can teach us about malaria. Am. J. Hum. Genet. *77*, 171–192.
14. Laval, G., Peyrégne, S., Zidane, N., Harmant, C., Renaud, F., Patin, E., Prugnolle, F., and Quintana-Murci, L. (2019). Recent Adaptive Acquisition by African Rainforest Hunter-Gatherers of the Late Pleistocene Sickle-Cell Mutation Suggests Past Differences in Malaria Exposure. Am. J. Hum. Genet. *104*, 553–561.
15. Louicharoen, C., Patin, E., Paul, R., Nuchprayoon, I., Witoonpanich, B., Peerapittayamongkol, C., Casademont, I., Sura, T., Laird, N.M., Singhasivanon, P., et al. (2009). Positively selected G6PD-Mahidol mutation reduces Plasmodium vivax density in Southeast Asians. Science *326*, 1546–1549.
16. Band, G., Rockett, K.A., Spencer, C.C., Kwiatkowski, D.P.; and Malaria Genomic Epidemiology Network (2015). A novel locus of resistance to severe malaria in a region of ancient balancing selection. Nature *526*, 253–257.
17. Shriner, D., and Rotimi, C.N. (2018). Whole-Genome-Sequence-Based Haplotypes Reveal Single Origin of the Sickle Allele during the Holocene Wet Phase. Am. J. Hum. Genet. *102*, 547–556.
18. Bos, K.I., Harkins, K.M., Herbig, A., Coscolla, M., Weber, N., Comas, I., Forrest, S.A., Bryant, J.M., Harris, S.R., Schuenemann, V.J., et al. (2014). Pre-Columbian mycobacterial genomes reveal seals as a source of New World human tuberculosis. Nature *514*, 494–497.
19. Sabin, S., Herbig, A., Vågene, A.J., Ahlström, T., Bozovic, G., Arcini, C., Kühnert, D., and Bos, K.I. (2020). A seventeenth-century Mycobacterium tuberculosis genome supports a Neolithic emergence of the Mycobacterium tuberculosis complex. Genome Biol. *21*, 201.
20. Wolfe, N.D., Dunavan, C.P., and Diamond, J. (2007). Origins of major human infectious diseases. Nature *447*, 279–283.
21. Mathieson, I. (2020). Human adaptation over the past 40,000 years. Curr. Opin. Genet. Dev. *62*, 97–104.
22. Jostins, L., Ripke, S., Weersma, R.K., Duerr, R.H., McGovern, D.P., Hui, K.Y., Lee, J.C., Schumm, L.P., Sharma, Y., Anderson, C.A., et al.; International IBD Genetics Consortium (IIBDGC) (2012). Host-microbe interactions have shaped the genetic architecture of inflammatory bowel disease. Nature *491*, 119–124.
23. Beecham, A.H., Patsopoulos, N.A., Xifara, D.K., Davis, M.F., Kemppinen, A., Cotsapas, C., Shah, T.S., Spencer, C., Booth, D., Goris, A., et al.; International Multiple Sclerosis Genetics Consortium (IMSGC); Wellcome Trust Case Control Consortium 2 (WTCCC2); and International IBD Genetics Consortium (IIBDGC) (2013). Analysis of immune-related loci identifies 48 new susceptibility variants for multiple sclerosis. Nat. Genet. *45*, 1353–1360.
24. Auton, A., Brooks, L.D., Durbin, R.M., Garrison, E.P., Kang, H.M., Korbel, J.O., Marchini, J.L., McCarthy, S., McVean, G.A., Abecasis, G.R.; and 1000 Genomes Project Consortium (2015). A global reference for human genetic variation. Nature *526*, 68–74.
25. Beaumont, M.A., Zhang, W., and Balding, D.J. (2002). Approximate Bayesian computation in population genetics. Genetics *162*, 2025–2035.
26. Gravel, S., Henn, B.M., Gutenkunst, R.N., Indap, A.R., Marth, G.T., Clark, A.G., Yu, F., Gibbs, R.A., Bustamante, C.D.; and 1000 Genomes Project (2011). Demographic history and rare allele sharing among human populations. Proc. Natl. Acad. Sci. USA *108*, 11983–11988.
27. Skoglund, P., and Mathieson, I. (2018). Ancient Genomics of Modern Humans: The First Decade. Annu. Rev. Genomics Hum. Genet. *19*, 381–404.
28. Lazaridis, I., Patterson, N., Mittnik, A., Renaud, G., Mallick, S., Kirsanow, K., Sudmant, P.H., Schraiber, J.G., Castellano, S., Lipson, M., et al. (2014). Ancient human genomes suggest three ancestral populations for present-day Europeans. Nature *513*, 409–413.
29. Haak, W., Lazaridis, I., Patterson, N., Rohland, N., Mallick, S., Llamas, B., Brandt, G., Nordenfelt, S., Harney, E., Stewardson, K., et al. (2015). Massive migration from the steppe was a source for Indo-European languages in Europe. Nature *522*, 207–211.
30. Albers, P.K., and McVean, G. (2020). Dating genomic variants and shared ancestry in population-scale sequencing data. PLoS Biol. *18*, e3000586.
31. Kay, G.L., Sergeant, M.J., Zhou, Z., Chan, J.Z.M., Millard, A., Quick, J., Szikossy, I., Pap, I., Spigelman, M., Loman, N.J., et al. (2015). Eighteenth-century genomes show that mixed

infections were common at time of peak tuberculosis in Europe. Nat. Commun. *6*, 6717.

32. Günther, T., and Nettelblad, C. (2019). The presence and impact of reference bias on population genomic studies of prehistoric human populations. PLoS Genet. *15*, e1008302.
33. Landrum, M.J., Lee, J.M., Benson, M., Brown, G.R., Chao, C., Chitipiralla, S., Gu, B., Hart, J., Hoffman, D., Jang, W., et al. (2018). ClinVar: improving access to variant interpretations and supporting evidence. Nucleic Acids Res. *46* (D1), D1062–D1067.
34. Miller, H.K., Schwiesow, L., Au-Yeung, W., and Auerbuch, V. (2016). Hereditary Hemochromatosis Predisposes Mice to Yersinia pseudotuberculosis Infection Even in the Absence of the Type III Secretion System. Front. Cell. Infect. Microbiol. *6*, 69.
35. Silva, N.M., Rio, J., and Currat, M. (2017). Investigating population continuity with ancient DNA under a spatially explicit simulation framework. BMC Genet. *18*, 114.
36. Olalde, I., Brace, S., Allentoft, M.E., Armit, I., Kristiansen, K., Booth, T., Rohland, N., Mallick, S., Szécsényi-Nagy, A., Mittnik, A., et al. (2018). The Beaker phenomenon and the genomic transformation of northwest Europe. Nature *555*, 190–196.
37. Mathieson, S., and Mathieson, I. (2018). FADS1 and the Timing of Human Adaptation to Agriculture. Mol. Biol. Evol. *35*, 2957–2970.
38. François, O., and Jay, F. (2020). Factor analysis of ancient population genomic samples. Nat. Commun. *11*, 4661.
39. Narasimhan, V.M., Patterson, N., Moorjani, P., Rohland, N., Bernardos, R., Mallick, S., Lazaridis, I., Nakatsuka, N., Olalde, I., Lipson, M., et al. (2019). The formation of human populations in South and Central Asia. Science *365*, 365.
40. Skourtanioti, E., Erdal, Y.S., Frangipane, M., Balossi Restelli, F., Yener, K.A., Pinnock, F., Matthiae, P., Özbal, R., Schoop, U.D., Guliyev, F., et al. (2020). Genomic History of Neolithic to Bronze Age Anatolia, Northern Levant, and Southern Caucasus. Cell *181*, 1158–1175.e28.
41. O'Neill, M.B., Shockey, A., Zarley, A., Aylward, W., Eldholm, V., Kitchen, A., and Pepperell, C.S. (2019). Lineage specific histories of Mycobacterium tuberculosis dispersal in Africa and Eurasia. Mol. Ecol. *28*, 3241–3256.

The American Journal of Human Genetics, Volume *108*

Supplemental data

Human ancient DNA analyses reveal the high burden of tuberculosis in Europeans over the last 2,000 years

Gaspard Kerner, Guillaume Laval, Etienne Patin, Stéphanie Boisson-Dupuis, Laurent Abel, Jean-Laurent Casanova, and Lluis Quintana-Murci

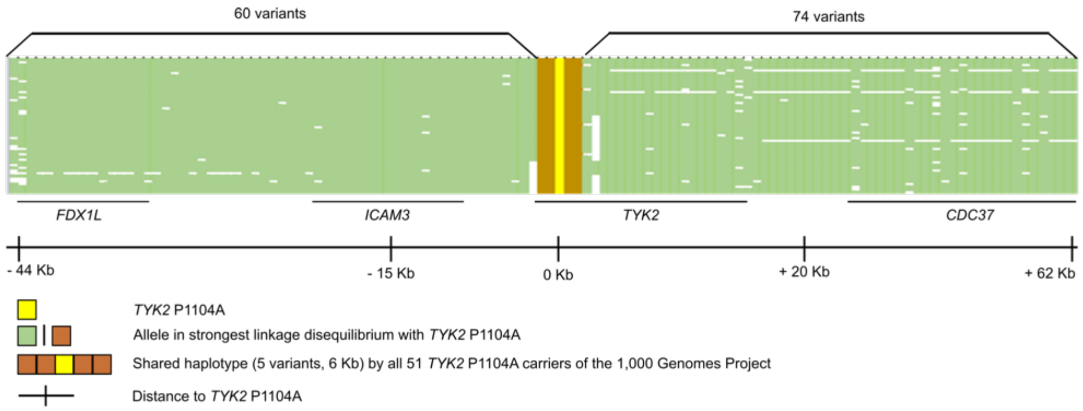

Figure S1. Founder effect for *TYK2* P1104A. Haplotypes (rows) for all 51 European (N=29), European-American (N=14), South Asian (N=6) and African-American (N=2) carriers of the *TYK2* P1104A allele of the 1,000 Genomes Project.[1] Variants (columns) outside a 6 Kb region around *TYK2* P1104A are colored according to whether the individual carries (green) or not (white) the allele in strongest linkage disequilibrium with *TYK2* P1104A for all biallelic sites with >1% frequency in 1,000 Genomes Project. All 51 heterozygous carriers of *TYK2* P1104A share a 6 kb-long haplotype composed of the variant itself, two variants upstream and two downstream it, which are the closest variants to *TYK2* P1104A, suggesting a worldwide founder effect.

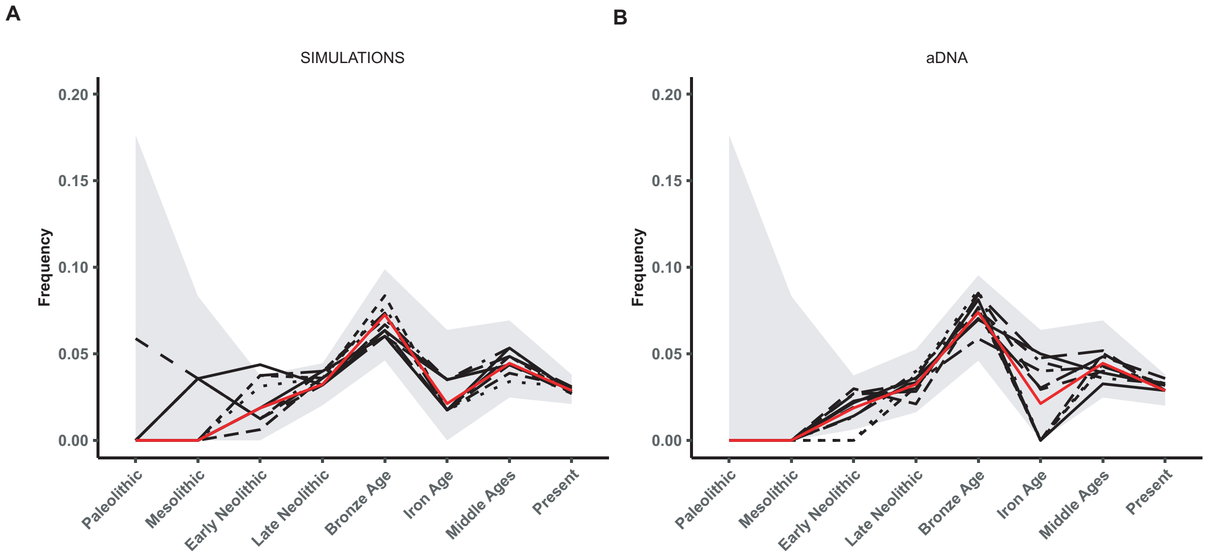

Figure S2. Top ten simulated or real aDNA variants best fitting the *TYK2* P1104A frequency trajectory. Top ten variants best fitting the observed data for (**A)** simulated variants under the proposed demographic model, with (uniformly distributed T_{onset} <10,000) or without negative selection, or (**B)** real aDNA data. The data is based on either 1,000,000 simulations or 1,000,000 randomly sampled aDNA variants. The red trajectory is that of *TYK2* P1104A. Uncertainty of the estimation is represented for *TYK2* P1104A frequencies as in **Figure 1**.

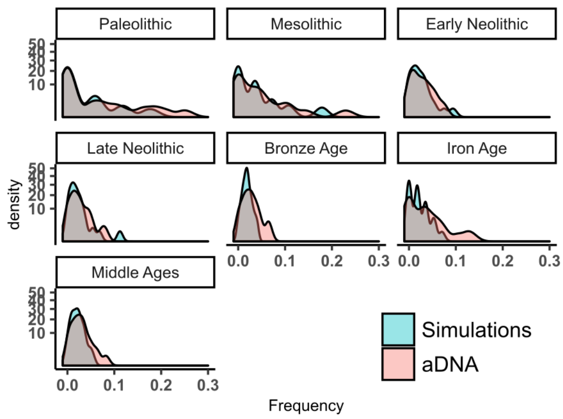

Figure S3. Matching between randomly simulated and real aDNA variants across different epochs. Densities of frequency for 500 randomly sampled simulated neutral or real aDNA variants with 3% frequency among present-day Europeans, for all seven (pre-) historical epochs. The y-axis is in root square scale for better visualization.

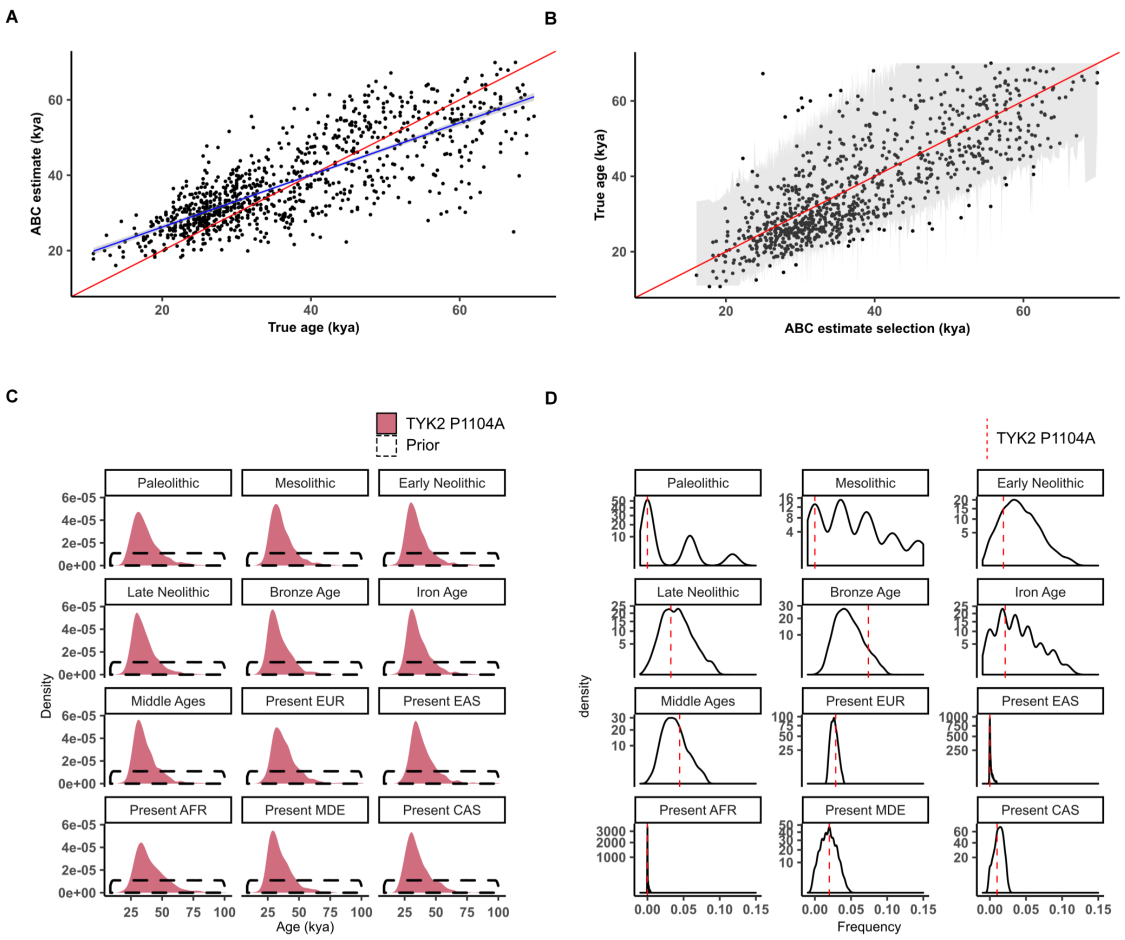

Figure S4. Model accuracy to assess the age of the *TYK2* P1104A mutation. **(A)** Leave-one-out cross validation model for the estimation of age, based on simulations in which the variant exists in present-day Europe, for N=1,000 replicates. The diagonal red line corresponds to the identity function and the blue line is the linear regression estimation on the data. True ages until 70,000 years are represented. True old (>50,000 years) ages are more inaccurately estimated than young (<50,000 years) ages. **(B)** The grey area indicates the range of 95% CIs (up and down bounds) for each ABC estimation of the age of mutation (ranked in ascendant order, *x*-axis). Black dots indicate the true age values for each corresponding ABC estimation, as in (A). For a 95% confidence interval, 96% of the estimated confidence intervals contain the true value. **(C)** Posterior distributions for the age of the *TYK2* P1104A mutation (as in **Figure 2B**), with each panel corresponding to the estimated distribution after removing one summary statistic (label on top) at a time. **(D)** Density distribution for each summary statistic (label on top) using only the simulations best fitting our data with selection and T_{onset} <10,000. Vertical dashed lines indicate the true value for *TYK2* P1104A. The y-axis is in root square scale for better visualization.

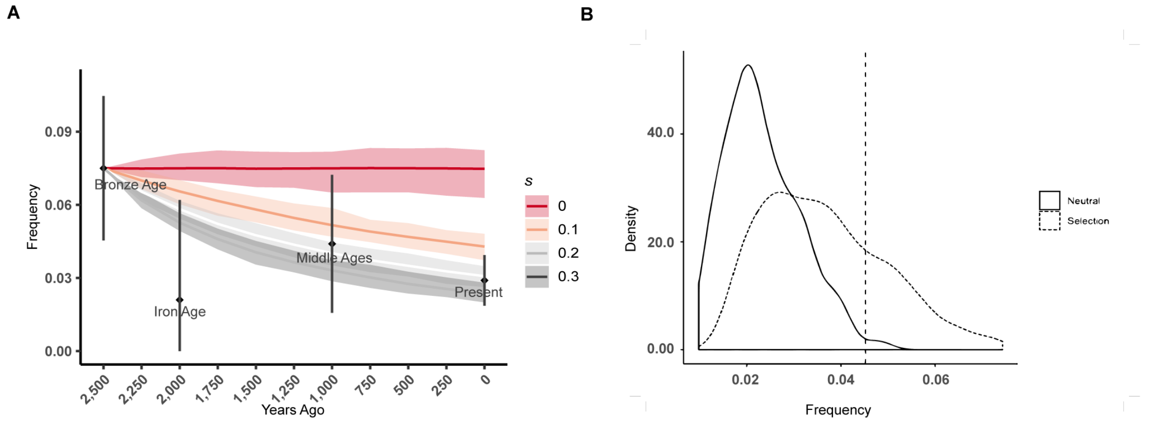

Figure S5. Unmatched frequency trajectory between neutrally-simulated variants and *TYK2* P1104A. **(A)** Average population frequency trajectories of 1,000 simulated variants from the end of the Bronze Age to the present, under the proposed demographic model, with an initial population frequency of 7.5% corresponding to that of P1104A 2,500 ya and a constant effective population sample size of 10,000, assuming neutrality (s = 0) or negative selection (s = 0.1, s = 0.2, s = 0.3). Colored areas indicate the 95% CI of the simulated frequencies at the population level at each generation. Black diamonds represent the P1104A frequency obtained from aDNA at each epoch since the Bronze Age (black bars indicate the confidence interval for the estimated frequency obtained from the aDNA sample, as in **Figure 1B**). **(B)** Distribution of the frequency variation between the Bronze Age and the present in Europe, for neutrally (solid line) or negatively-selected (dotted) simulated variants best fitting the observed data, using 1,000,000 simulations, each. The vertical dashed line represents the real frequency decrease observed for *TYK2* P1104A.

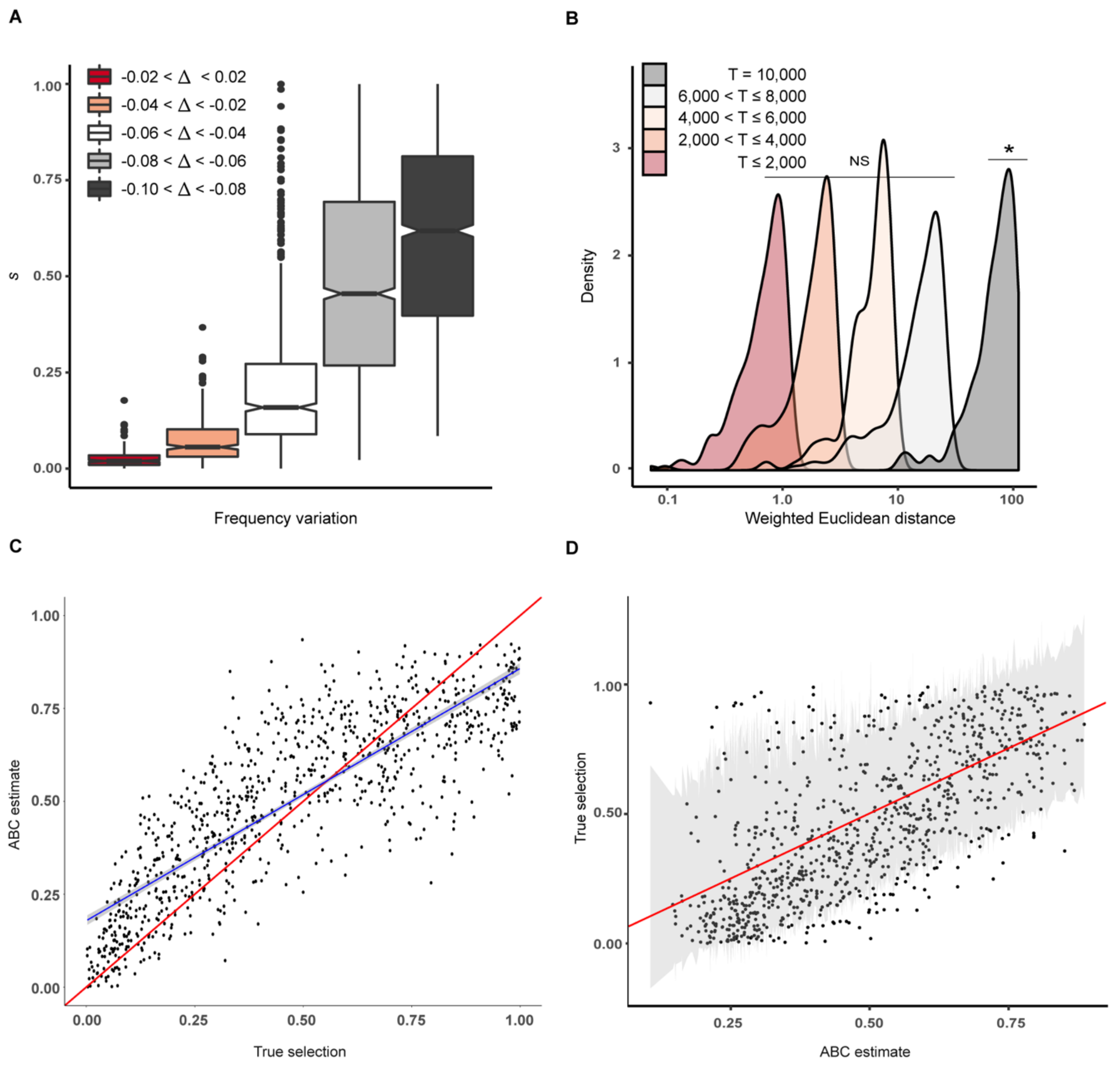

Figure S6. Model accuracy to assess negative selection. **(A)** Distribution of the estimated negative selection coefficient (*s*), as a function of the absolute variation in frequency (Δ) in a frame of 3,000 years. Δ is negative for variants that decreased in frequency over time. **(B)** Distribution of the weighted Euclidean distances as in **Figure 3B**, with T_{onset} = 10,000 ya (dark grey), 6,000 < T_{onset} ≤ 8,000 (light grey), 4,000 < T_{onset} ≤ 6,000 (light peach), 2,000 < T_{onset} ≤ 4,000 (dark peach), or 250 < T_{onset} ≤ 2,000 (red), using European summary statistics from the Late Neolithic epoch onwards. For each T_{onset} interval, 2,000,000 simulations were used. A two-sampled Hotelling's T-squared test was used to assess whether the simulations best fitting our data for the early onset model (T_{onset} = 10,000; $p = 5.4 \times 10^{-4}$; *) or for that with variable onset of selection model ($p = 0.09$; NS: non-significant) were drawn from the same distribution than that of *TYK2* P1104A. The x-axis is in log-scale for a better visualization. **(C)** Leave-one-out cross validation (N=1,000) for the strength of negative selection, as in **Figure S4A**. The analysis was obtained for simulated variants best fitting the frequency of *TYK2* P1104A at the Bronze age, with T_{onset} <10,000 and 10,000,000 simulations. **(D)** The grey area indicates the range of 95% CIs (up and down bounds) for each ABC estimation of the selection coefficient (ranked in ascendant order, *x*-axis). Black dots indicate the true values for each corresponding ABC estimation, as in (C). For a 95% confidence interval, 93% of the confidence intervals contain the true value.

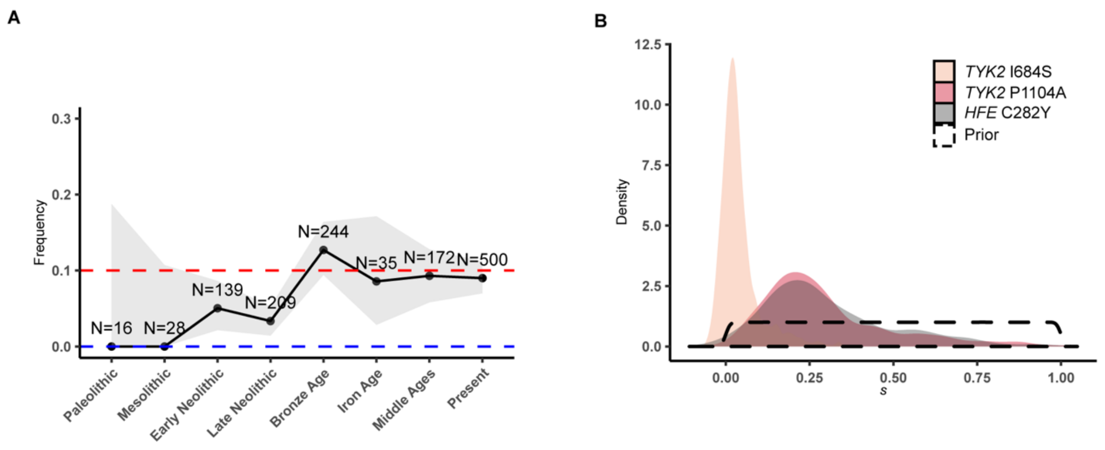

Figure S7. Selection estimations for *TYK2* I684S used for comparative purposes. **(A)** Frequency trajectory for *TYK2* I684S, as in **Figure 1B**. **(B)** Posterior distributions for the strength of negative selection for *TYK2* I684S (beige) and *TYK2* P1104A (red), as in **Figure 3A**.

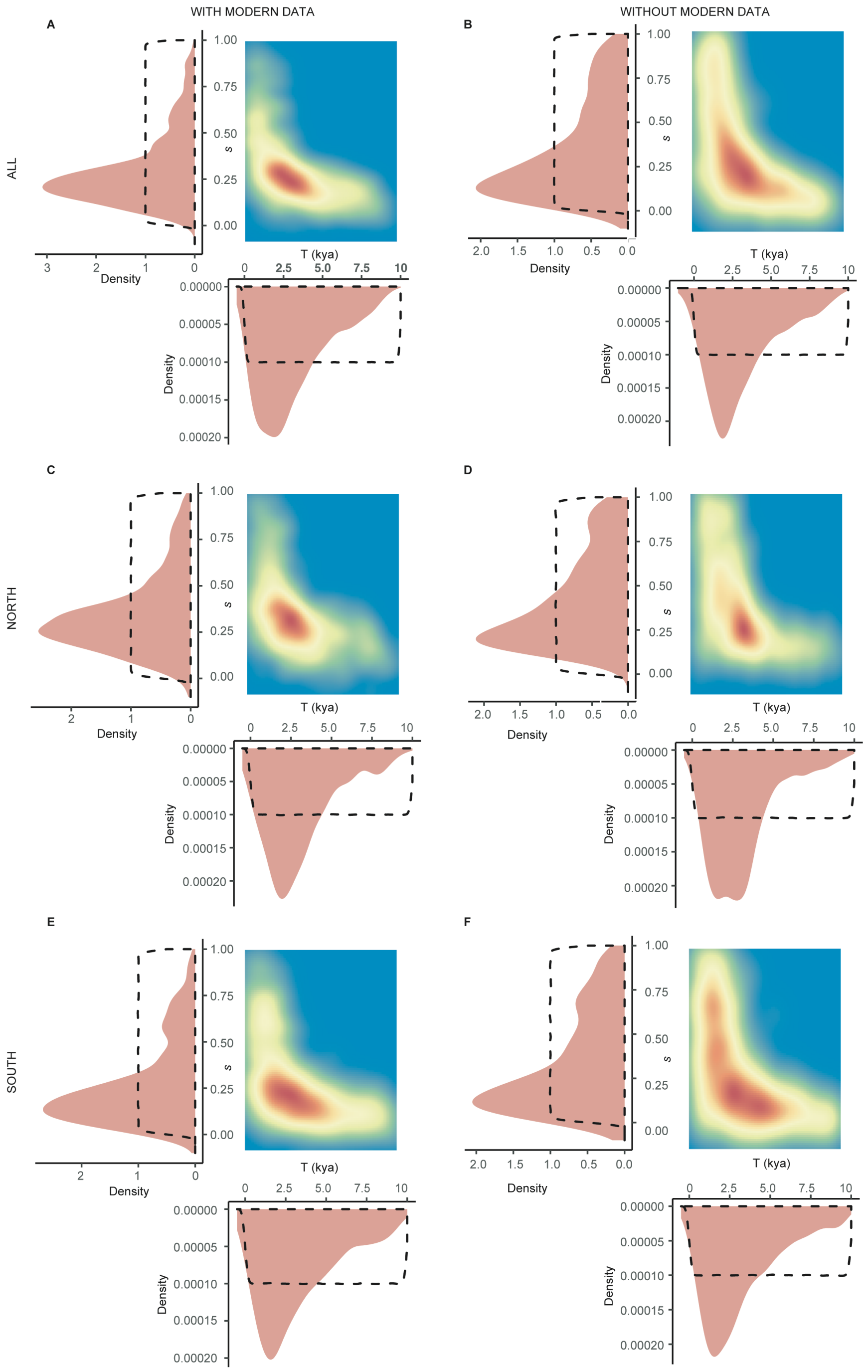

Figure S8. Estimation of the onset and strength of negative selection for *TYK2* P1104A in the case of population stratification or read-bias. Posterior distributions for the negative selection coefficient and the onset of negative selection as in **Figure 3A** for the European population as defined in the main text **(A)** including (*s*: mode = 0.21; 95% CI: [0.06-0.82];) or **(B)** excluding (*s*: mode = 0.13; 95% CI: [0.02-0.94]) modern data, the Northern European population (Supplemental Methods) **(C)** including (*s*: mode = 0.24; 95% CI: [0.02-0.87]) or **(D)** excluding (*s*: mode = 0.21; 95% CI: [0.10-1]) modern data, and the Southern European population (Supplemental Methods) **(E)** including (*s*: mode = 0.13; 95% CI: [1.6×10^{-4}-0.81]) or **(F)** excluding (*s*: mode = 0.11; 95% CI: [0-0.94]) modern data. In all cases considered, we found similar estimations for *s* (higher for northern Europeans, intermediate for the merge of all European populations, and slightly lower for southern Europeans**)**. This suggests that our estimation is not biased due to a systematic read-bias in the aDNA samples.

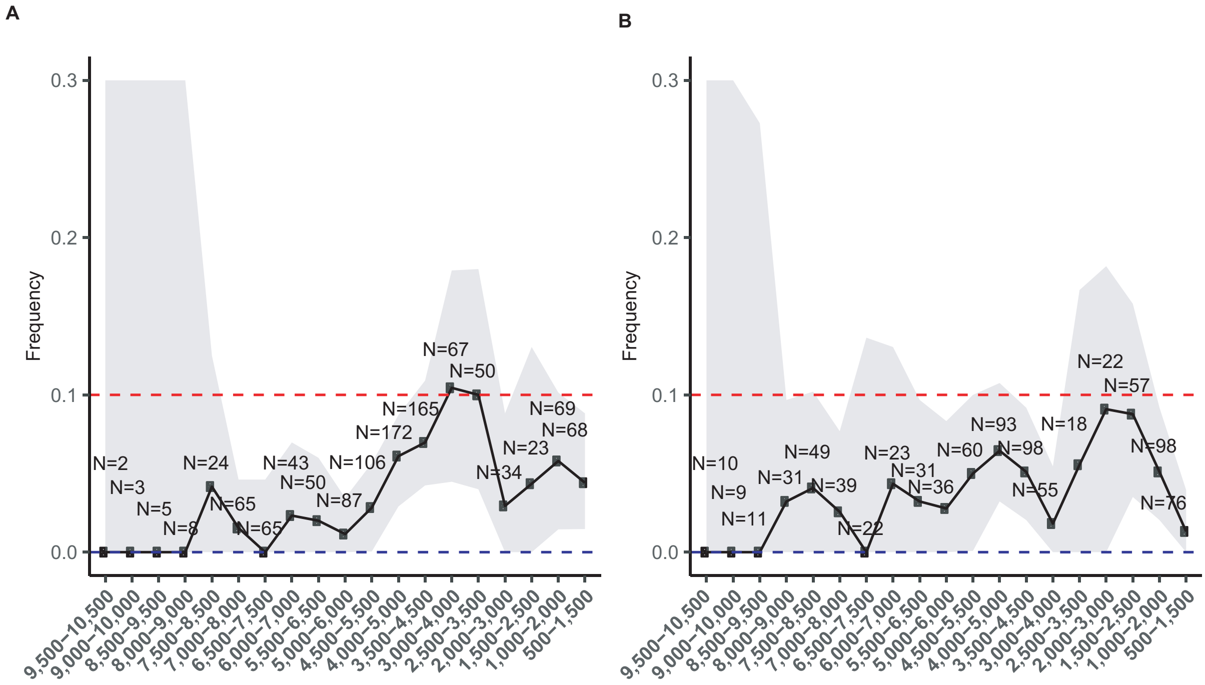

Figure S9. Evolutionary history of the TB-associated *TYK2* P1104A variant in Northern or Southern Europe. **(A)** Northern (with high Steppe-ancestry) or **(B)** Southern **(**low Steppe-ancestry) European frequency trajectories for the *TYK2* P1104A variant over the last 10,000 years for bins of 1,000 years and sliding windows of 500 years as in **Figure 1A**.

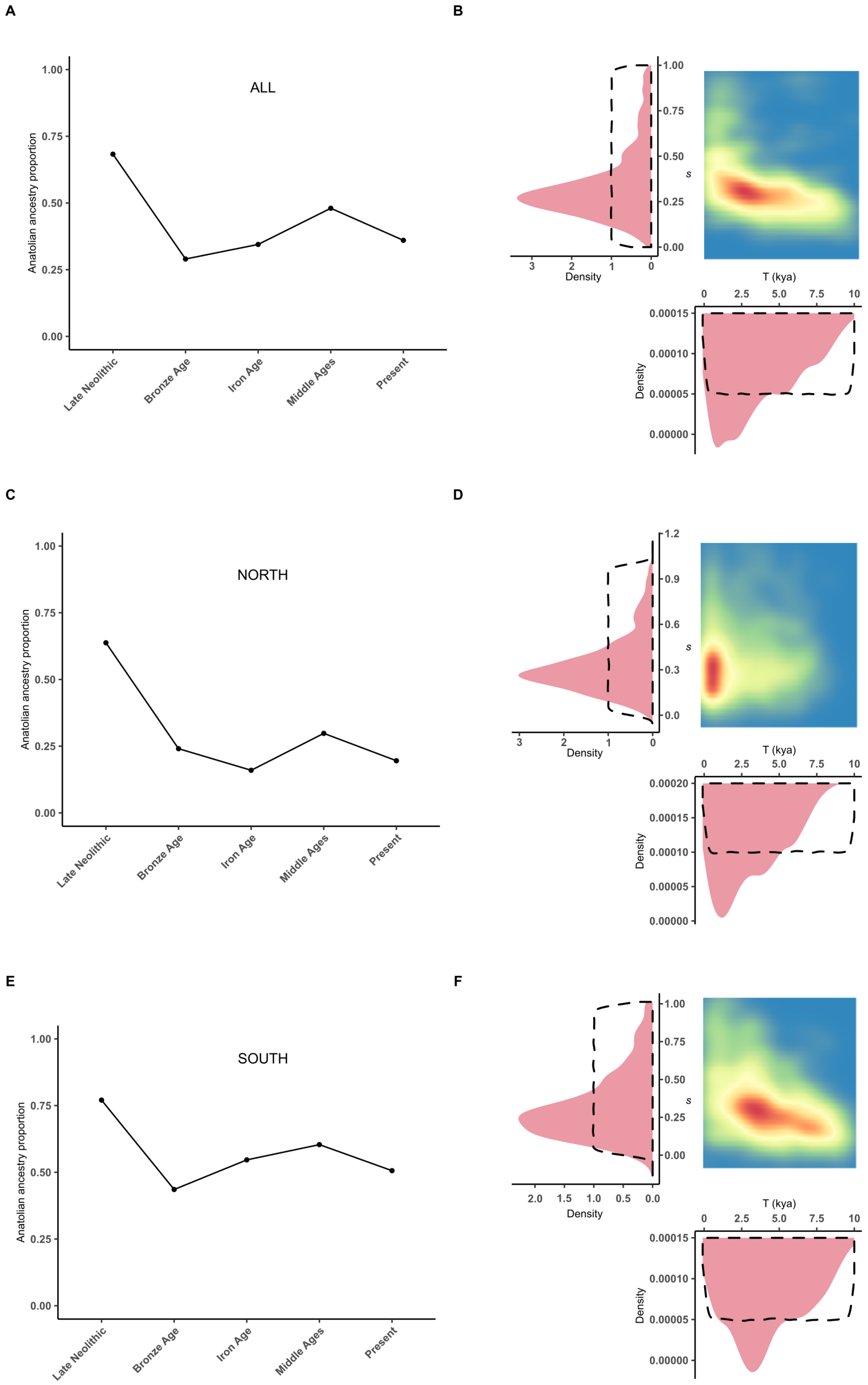

Figure S10. Left part of the figure: Anatolian ancestry trajectory on the basis of **(A)** the full dataset of available aDNA genomes, **(C)** only northern Europeans and **(E)** only southern Europeans. Right part: Estimation of the onset and strength of negative selection for *TYK2* P1104A when accounting for ancestry variations across epochs for **(B)** the full dataset, **(D)** only northern Europeans and **(F)** only southern Europeans. Posterior distributions for the negative selection coefficient and the onset of negative selection are shown as in **Figure 3A**.

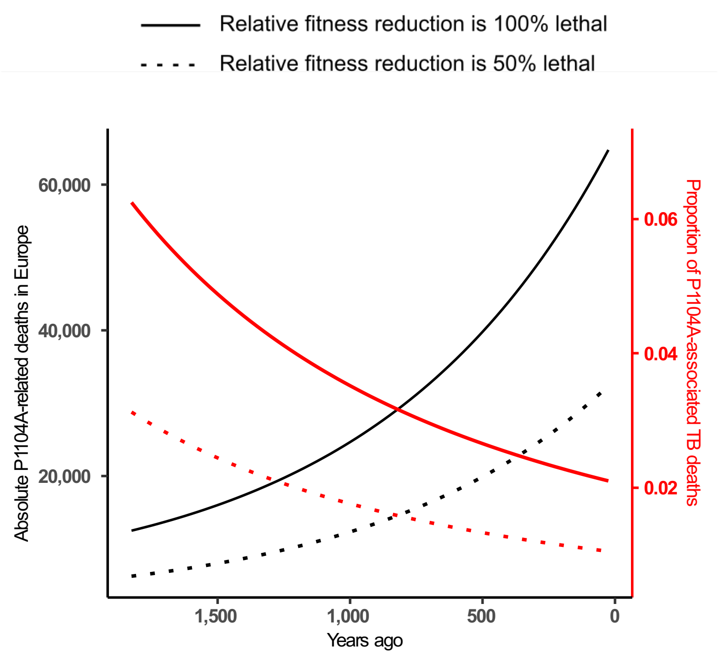

Figure S11. *TYK2* P1104A-related deaths or TB deaths in Europe during the common era. Absolute number of *TYK2* P1104A-related deaths (black) or proportion of TB-related deaths due to *TYK2* P1104A (red), assuming the relative fitness reduction (as estimated by our approach) to be 100% (solid) or 50% (dotted) lethal.

Table S1. Collection of 1,013 ancient genomes covering a time transect from the Mesolithic period to the Middle Ages.

Uploaded as a separate excel file ‘Table_S1.xlsx’

Table S2. Parameters and value ranges to calibrate the updated demographic model

Parameter	Minimum value	Maximum value
Split AFR-NonAFR (*kya*)	60	80
Ne (NonAFR)	2,000	3,000
Split WestNonAFR-EastNonAFR (*kya*)	35	45
Ne (WestNonAFR)	3,000	4,000
Split EUR-Anatolia/Steppe (*kya*)	15	25
Ne (Anatolia/Steppe)	= Ne WestNonAFR	= Ne WestNonAFR
Pulse-Anatolia (*kya*)	8	10
Pulse-Steppe (*kya*)	4	6
Intensity Pulse-Anatolia	0	100
Intensity Pulse-Steppe	0	100

Note: Minimum and maximum values define the accepted range for each parameter. A uniform distribution was used to drawn values from these ranges. *Split* stands for divergence time, *Ne* for effective population size, *Pulse* for the event of instantaneous migration (accounting for x% of migrants of the source population, where x is given by the intensity of the pulse), and *kya* for thousands of years.

Table S3. Values for the empirical summary statistics

Summary Statistic	Value	Uncertainty range[a] (P1104A frequencies)
Paleolithic	0	[0-0.171]
Mesolithic	0	[0-0.081]
Early Neolithic	0.019	[0-0.040]
Late Neolithic	0.032	[0.010-0.054]
Bronze Age	0.076	[0.041-0.107]
Iron Age	0.021	[0-0.062]
Middle Ages	0.045	[0.016-0.074]
Present Europe	0.029	[0.018-0.039]
Present Middle East	0.021	-
Present Central Asia	0.010	-
Present Sub-Saharan Africa	0	-
Present East Asia	0	-
Late Neolithic Anatolian Ancestry	0.683	-
Bronze Age Anatolian Ancestry	0.290	-
Iron Age Anatolian Ancestry	0.345	-
Middle Ages Anatolian Ancestry	0.480	-
Present Europe Anatolian Ancestry	0.360	-

[a] Normal approximation of the 95% CI assuming a binomial distribution for the estimated frequency. For frequencies at 0 we assumed the existence of one carrier to draw CIs, a conservative scenario for the uncertainty range.

Table S4. Simulation sets

	**Neutral**	**Early Onset**	**Full**[a]
T_{age}	$\mathcal{U}$[8.5-100] kya	$\mathcal{U}$[8.5-100] kya	$\mathcal{U}$[8.5-100] kya
s	FIXED, $s = 0$	$\mathcal{U}$[0-1]	$\mathcal{U}$[0-1]
T_{onset}	NA[b]	FIXED, $T_{onset} = 10$ kya	$\mathcal{U}$[0.5-10] kya
# Simulations	1,000,000	1,000,000	10,000,000
Tolerance rate[c]	0.0001	0.0001	0.0001
# Best fitting simulations	100	100	1,000

Note: T_{age}: age of the mutation; s: selection coefficient; T_{onset}: onset of Selection. $\mathcal{U}$ stands for Uniform prior distribution. FIXED stands for fixed value share across all simulations. NA stands for "not applicable".

[a] Simulations underlying the Full set were conducted either accounting or not for ancestry, leading to two sets of 10,000,000 simulations. [b] In the Neutral set of simulations, the onset of selection is undefined since there is no selection. [c] The ABC tolerance rate defines the fraction of simulations that defines the best fitting simulations, i.e., the percent of simulations with the lowest Euclidean distances to the data.

Table S5. Selection estimations for the top 10 variants of Figure S2B.

ID variant	Sel (s)	Sel 95% CI_l	Sel 95% CI_u	Onset	Onset 95% CI_l	Onset 95% CI_u
rs10509135	0.34	0.17	1	1,334	500	8,231
rs10810211	0.21	0.05	0.91	2,746	500	9,665
rs11839433	0.22	0.05	0.90	2,568	500	9,362
rs17065399	0.21	0.05	0.89	2,199	500	9,132
rs75948696	0.30	0.12	0.99	1,626	500	8,635
rs4535615	0.27	0.10	0.97	2,033	500	9,009
rs75379571	0.22	0.07	0.92	2,498	500	9,332
rs7593276	0.22	0.06	0.90	2,156	500	9,005
rs9506920	0.25	0.10	0.95	2,087	500	8,923
rs73664354	0.21	0.06	0.92	2,721	500	8,729

Note: Estimations have been conducted with ABC accounting for ancestry as presented in section ‘Accounting for ancestry in the ABC estimation’.

Supplemental Methods

Ancient DNA and present-day samples

We analyzed 1,013 aDNA genomes (**Table S1)** that: (i) originate from burial sites of western Eurasia (-9< longitude (°) <42.5 and 36< latitude (°) <70.1), (ii) are covered at the *TYK2* P1104 position, and (iii) were retrieved from the V42.4: March 1 2020 release at https://reich.hms.harvard.edu/downloadable-genotypes-present-day-and-ancient-dna-data-compiled-published-papers. Ancient individuals were treated as pseudo-haploid (i.e. carry only one allele) because most samples are low coverage. We manually removed duplicated samples that were explicitly annotated. For many of these samples, 1,233,013 sites were genotyped using an in-solution capture method, which includes the *TYK2* P1104 variant position, while those with whole-genome shotgun sequence data were genotyped at the same set of sites. We also used the *TYK2* P1104A frequencies observed in Europeans, East Asians and Africans from the 1,000 Genomes Project,[1] as well as from 336 Central Asians and 156 Middle Easterners.[2,3]

TYK2 P1104A frequency trajectory

Ancient genomes were grouped by major time periods divided into well-accepted cultural eras: the Paleolithic (51,000 [oldest data in the database] - 12,000 ya), the Mesolithic (12,000 ya - 8,500 ya), the Early (8,500 ya - 6,500 ya) and the Late (6,500 ya - 4,500 ya) Neolithic, the Bronze Age (4,500 ya - 2,800 ya), the Iron Age (2,800 ya - 2,000 ya) and the Middle Ages (2,000 ya - 500 ya). Data was scarce for the Paleolithic (N = 17), the Mesolithic (N = 36) and the Iron Age (N = 47), while estimations for other time periods were obtained from at least 100 aDNA samples each, the Bronze Age being the most covered (N = 304). We also checked for the existence of annotated first-degree familial relationships among the 1,013 ancient genomes of our dataset. We found that the exclusion of all but one individual of each related group (N = 2 exclusions for the Mesolithic, N = 4 for the Early Neolithic, N = 11 for the Late Neolithic, N = 17 for the Bronze Age, N = 0 for the Iron Age and N = 8 for the Middle Ages) had no effects on frequency estimations of P1104A at each epoch, and, more importantly, that no ancient P1104A carriers were related to another individual of the cohort.

Simulated *TYK2* P1104A frequency trajectory

Computer simulations of an allele evolving under a given demographic model were carried out using SLiM 3 (ref.[4]). Because SLiM is a forward-in-time simulator,[4] the computation times, which depend on both the effective population size N and the number of generations t

considered, are large for the model we aimed to simulate. We thus rescaled effective population sizes and times according to N_e/λ and t/λ, with λ = 10 (ref.[4,5]). We assumed a single origin of the *TYK2* P1104A mutation at different times (see section 'Age and origin estimation for the *TYK2* P1104A mutation'). To simulate aDNA, we randomly sampled simulated diploid individuals at generations corresponding to the radiocarbon calibrated estimations for the age of the 1,013 available ancient genomes. For each sampled individual, we then randomly selected an allele to generate pseudo-haploid data, mirroring the observed pseudo-haploid aDNA data used in this study. Additionally, simulated European, East Asian, African, Central Asian and Middle Easterner present-day individuals were randomly drawn at the last generation of the simulated demography, accounting for the sample sizes of the observed data (see section 'Ancient DNA and present-day samples'). The simulated allele frequency trajectory was therefore computed from simulated pseudo-haploid aDNA and diploid modern data, as it was done for the observed data.

Simulated demographic model

The allele frequency trajectory was obtained and assessed under a classical model for which demographic parameters, including divergence times, effective population sizes, migration rates and exponential growth of the three considered populations (ancestral African population, and West and East Eurasians) were available.[6] Because this model neglects major migratory events inferred with aDNA data,[7] we expanded the model to include the arrival of early farmers from the Anatolian peninsula ~8,500 ya and the subsequent migrations associated with Eurasian steppe-related populations ~4,500 ya.[8-14] In the updated model, divergence times between Europeans and Middle Easterners and Central Asians were defined as nuisance parameters (**Table S2**). The range for these parameters were based on recent estimates from whole-genome sequences of individuals from the Human Genome Diversity Project (HGDP).[15] Migrations of Middle Easterners and Central Asians into Europe were modelled as pulses occurring at varying time points, with rates ranging from 0 to 100%.

ABC estimation

We used an approximate Bayesian computation (ABC) approach[16,17] to estimate the age (T_{age}), the strength (s) and the onset (T_{onset}) of negative selection acting upon the *TYK2* P1104A mutation. Parameters were estimated using computer simulations best fitting the observed data. Simulated and empirical data were described by a vector of K summary statistics, which were used to fit the observed data. For each parameter, posterior

distributions, point estimates (in this case, posterior mode) and the 95% CIs were obtained from accepted simulated parameter values, that is, parameter values obtained from simulations where summary statistics are in close match with those of the empirical data. We used the ‘abc’ R package and the standard ABC method,[18,19] for which the accepted simulated parameters were subsequently adjusted by local linear regression (method = “Loclinear” in the ‘abc’ package). In this study, summary statistics included allele frequencies from simulated frequency trajectories. We modified the R package to allow matches between simulated and empirical summary statistics by means of weighted (based on sample sizes by epochs) Euclidean distances. Parameter estimations were obtained from 10,000,000 simulations drawn with predefined prior distributions for each parameter (priors are specified in the sections ‘Age and origin estimation for the *TYK2* P1104A mutation’ and ‘Onset and strength of negative selection estimation for *TYK2* P1104A’). Accepted parameters were those of simulations providing the 0.01% lowest Euclidean distances between observed and simulated statistics (the ‘abc’ parameter ‘tol’ was set to be equal to 0.0001). Thus, ABC point estimates and 95% CIs for the age of the mutation, the strength and onset of selection (see below) were computed from the 1,000 best fitting simulations (0.01% of the total 10,000,000).

Age and origin estimation for the *TYK2* P1104A mutation

Age estimation (T_{age}) was obtained by ABC, using simulated frequency trajectories with or without selection (see section ‘Onset and strength of negative selection estimation for *TYK2* P1104A’). As summary statistics, we considered all $K = 12$ available frequency estimations from the frequency trajectory, including ancient (from Paleolithic to Middle Ages) and modern DNA data (from Sub-Saharan Africa, Europe, Middle-East, Central-Asia and East-Asia) (**Table S3**). We removed archaic individuals (N=4, aged >40,000 ya) to the estimation of T_{age}, and assumed a uniform prior distribution for the age of the mutation over the last 100,000 year and a single origin for the *TYK2* P1104A mutation. Because the first occurrence of the allele in our dataset was observed 8,500 ya, we randomly sampled T_{age} from $\mathcal{U}[8.5\text{-}100]$ kya.

Onset and strength of negative selection estimation for *TYK2* P1104A

We assumed a relative fitness for each *TYK2* genotype of $w_{WT/WT} = 1$, $w_{WT/P1104A} = 1$, and $w_{P1104A/P1104A} = 1 - s$, consistent with experimental and epidemiological data suggesting a recessive effect of P1104A on disease risk.[20,21] In the analysis of selection (onset and

strength), we considered three simulation sets (**Table S4**). Consistent with previous dating of *M. tuberculosis* during the Neolithic,[22,23] we first explored the hypothesis of an early onset of negative selection. We performed 1,000,000 simulations assuming a continuous and constant negative selection on homozygous carriers starting 10,000 ya, by randomly sampling s from $\mathcal{U}[0\text{-}1]$ and fixing T_{onset} to 10,000 ya ("Early onset" set; **Table S4**). We then compared the early onset set with a second simulation set with more recent onsets of negative selection (varying from 10,000 ya to 500 ya, 250 years being the generation unit in our rescaled model). We performed 10,000,000 simulations randomly drawing s and T_{onset} from the uniform distributions $\mathcal{U}[0\text{-}1]$ and $\mathcal{U}[500\text{-}10{,}000]$, respectively ("Full" simulation set, Table S4). For this set, we performed ten times more simulations in order to better explore the parameter space, which is much larger in this case since T_{onset} also varies across simulations. In both cases, the allele was assumed to be neutral before the onset of selection. Here, a two-sampled Hotelling's T-squared test was used to assess whether the simulations best fitting the empirical data were drawn from the same distribution than that of *TYK2* P1104A (considering P1104A's trajectory as curves within its uncertainty frequency interval, **Table S3**). Finally, a third simulation set was conducted assuming a neutral evolution of the *TYK2* P1104A allele, by performing 1,000,000 simulations fixing s to 0 ("Neutral" simulation set, **Table S4**). This simulation set was compared to the "Full" set to assess the likelihood of negative selection in the evolution of the P1104A allele (see section 'Odds ratio (OR) computation').

The ABC estimations of s and T_{onset} were performed using the aforementioned "Full" set of 10,000,000 simulations and European summary statistics from the Late Neolithic epoch onwards (K=5; **Table S3**), as described in the main text. To account for population stratification, we considered a north-south geographical division for Europe. Specifically, we defined "northern Europe" to be the region east of 13°E and north of 45°N, or west of 13°E and north of 49°N, with "southern Europe" as the complement, as previously proposed.[11]

Because of the adopted rescaled model (see section 'Simulated *TYK2* P1104A frequency trajectory'), we used rescaled λs. Specifically, we used an iterative algorithm to obtain selection coefficients for the rescaled model, designed to provide expected trajectories of the selected allele under a non-rescaled model. Briefly, parameters of the model were multiplied by a factor λ (here $\lambda = 10$) to account for the rescaling process. However, if $s > 0.1$, $\lambda s > 1$, which lies outside the accepted range of values for selection coefficients ([0-1]). To compensate for this, we modified the dominance coefficient λh of the rescaled model for values of $s > 0.1$. We first observed the relationship between frequency changes from one

generation t to the next (t+1) for a biallelic locus with alleles ‘a’ and ‘A’. We assumed a relative fitness for each genotype of $P(r_t|AA)$ = 1, $P(r_t|aA)$= 1 - hs, and $P(r_t|aa)$ = 1 – s.

$$P_{t+1} = P(aa|r_t) + \frac{1}{2}P(aA|r_t),$$

where frequency of allele a at time t is denoted P_t (and that of time t+1, P_{t+1}), and r_t is the Boolean event of either contributing or not to the offspring pool at generation t+1. Also,

$$P(aa|r_t) = \frac{P(r_t|aa)P(aa)}{P(r_t|aa)P(aa) + P(r_t|aA)P(aA) + P(r_t|AA)P(AA)}$$
$$= \frac{(1-s)P_t^2}{(1-s)P_t^2 + 2(1-hs)P_t(1-P_t) + (1-P_t)^2}.$$

Repeating this process for the heterozygous genotype and adding accordingly, we obtain,

$$P_{t+1} = \frac{(1-s)\,P_t^2 + (1-hs)(1-P_t)}{(1-s)\,P_t^2 + 2(1-hs)P_t(1-P_t) + (1-P_t)^2}. \quad (1)$$

Now, simply using formula (1) we estimate λh_t, which is the dominance coefficient at generation t in the rescaled model, to accommodate for the true frequency at generation t, denoted P_t, assuming s >0.1. To do so, if t0 is the first generation in the rescaled model at which s >0.1, we repeat iteratively λ-1 times formula (1) starting with $P'_{t0} = P_{t0\times\lambda}$, s and h = 0, to obtain the expected frequency at generation t0+1, denoted P'_{t0+1}. Using known P'_{t0} and $\lambda s = -1$, we derive the value of λh_{t0} as follows,

$$\lambda h_{t0} = 1 - \frac{P_{t0+\lambda-1}(1-P'_{t0})^2}{(1-2P_{t0+\lambda-1})P_{t0}(1-P'_{t0})}.$$

Analogously, we obtained λh_t for all generations t for which s >0.1.

Odds ratio (OR) computation

To localize the origin of the P1104A mutation, we focused on the “Full” simulation set (**Table S4**), from which we obtained estimates for T_{age}. We compared the proportion of

variants arising in the common ancestors of West Eurasians (WE) in the 1,000 best fitting simulations with that of the remaining 99,999,000 simulations. Then, we computed an OR from the following 2 x 2 contingency table:

Origin / Simulations	Common Ancestor WE	Otherwise
**Best Fitting**	606	394
**Others**	1,806,191	8,192,809

We also provided evidence in favor of a negatively selected evolution for P1104A by comparing the "Full" with the "Neutral" simulation set (**Table S4**). We estimated, for each set, the proportion of best fitting simulations that had a frequency decrease at least as much as that of P1104A since the Bronze Age. We then computed an OR from the following 2 x 2 contingency table:

Frequency decrease / Simulations	At least as P1104A	Otherwise
**Best Fitting Full**	250	750
**Best Fitting Neutral**	1	99

The ORs, the 95% CI and the chi square *p*-value were computed using the R (version 3.6.0) package "epitools". The corresponding OR was computed as follow

OR = $\frac{250/(1000-250)}{1/(100-1)}$ = 33 (95% CI = [5-240], $p < 10^{-10}$).

Historical burden of TB in Europe due to homozygosity for *TYK2* P1104A

Using our point estimations for the onset and the strength of negative selection on *TYK2* P1104A, we sought to assess the historical number of *TYK2* P1104A-related deaths. This number was assessed following a number of assumptions: 25,000,000 individuals in Europe 2,000 ya,[24] a frequency of 0.05 for *TYK2* P1104A 2,000 ya, consistent with our aDNA data, and a previously defined exponential growth coefficient for Europeans,[6] leading to a realistic 520,000,000 Europeans today. The number of homozygotes at each generation (25 years) was obtained using Hardy-Weinberg equilibrium and the respective European sample size ($f^2\times$ sample size), the allele being in Hardy-Weinberg equilibrium in the current generation. We

also assumed that the relative 20% loss of fitness among homozygotes due to TB was either 100% or 50% lethal, so that 20% or 10% of *TYK2* P1104A homozygotes died at each generation due to TB. We further assessed the proportion of TB-related deaths due to *TYK2* P1104A assuming a constant mortality rate of TB of 800 in 100,000 until 200 ya.[25]

Factor Analysis

Factor analysis[26] was conducted on a merged data set consisting of 143,081 SNP genotypes for 363 present-day European individuals consisting mainly on the 1,000 Genomes Project individuals (IBS, TSI, GBR and FIN, available at the V42.4: March 1 2020 release) and 701 ancient samples from the aDNA dataset (see section 'Ancient DNA and present-day samples'). Factors are interpreted as principal components from principal component analysis but with temporal correction for present-day and ancient samples. The samples had ages less than 14,000 years old, and were chosen for their higher level of genomic coverage (>0.9X), as previously proposed.[26] Following this approach, we chose the drift parameter in order to remove the effect of time on the Kth factor (K = 4 here), where K is the number of ancestral groups considered.

Accounting for ancestry in the ABC estimation

We re-estimated the selection coefficient (*s*) and the onset of selection (T_{onset}) using the previously defined ABC approach, accounting for the observed ancestry proportions across epochs. To do so, we estimated ancestry proportions for ancient and modern samples by epoch, based on the admixture rates obtained from the previously described Factor Analysis,[26] using the 'ancestry_coefficients' function of the 'tfa' R (version 3.6.0) package. We used as European source populations either 21 Mesolithic hunter-gatherers (group ID 'Serbia_Mesolithic_IronGates') and 18 Anatolian (group ID 'Anatolia_N') farmers (until 5,000 ya) or the 18 Anatolian farmers and 1 herder (group ID 'Russia_Caucasus_EBA_Yamnaya') from the Steppes (after 5,000 ya) (**Table S1**), the proportion of Mesolithic hunter-gatherer ancestry at the population level being comparatively very low after the Bronze Age.[10]. We then summarized the estimations obtained for the Anatolian ancestry component as a frequency trajectory over time (**Table S3**).

Aside from the previously used summary statistics, we included to the ABC approach the average Anatolian ancestry proportions estimated at the Late Neolithic, the Bronze Age, the Iron Age, the Middle Ages and present times. To derive a simulated Anatolian ancestry trajectory of the sample, we added to the source Anatolian (MDE, **Figure 2B**) simulated

population a fixed marker variant that uniquely identifies individuals from this population, and that we linked to the variant under study (complete linkage disequilibrium between the simulated selected variant and the ancestry marker). We then used the frequency of the aforementioned marker variant in the EUR (**Figure 2B**) population to provide an estimation of the Anatolian ancestry proportion of the sample by epoch in Europe.

Reference bias in aDNA

The estimation of the allele frequency trajectory relies on the quality of the reads obtained from aDNA. In short-read mapping and in the process of in-solution enrichment, there may be a bias towards one of the two alleles present at a polymorphic site,[27] which usually favors the reference allele (the ancestral allele for the *TYK2* P1104 position). The combination of modern (without this issue) and aDNA data may therefore result in selection estimation bias, which, in the case of a bias favoring the reference allele, would result in an underestimation of s in our approach. We therefore removed modern data from our analysis and re-estimated s for northern, southern or all Europeans together.

Supplemental References

1. 1000 Genomes Project Consortium (2015). A global reference for human genetic variation. Nature *526*, 68-74.
2. Scott, E.M., Halees, A., Itan, Y., Spencer, E.G., He, Y., Azab, M.A., Gabriel, S.B., Belkadi, A., Boisson, B., Abel, L., et al. (2016). Characterization of Greater Middle Eastern genetic variation for enhanced disease gene discovery. Nat Genet *48*, 1071-1076.
3. Marchi, N., Mennecier, P., Georges, M., Lafosse, S., Hegay, T., Dorzhu, C., Chichlo, B., Segurel, L., and Heyer, E. (2018). Close inbreeding and low genetic diversity in Inner Asian human populations despite geographical exogamy. Sci Rep *8*, 9397.
4. Haller, B.C., and Messer, P.W. (2019). SLiM 3: Forward Genetic Simulations Beyond the Wright–Fisher Model. Mol Biol Evol *36*, 632-637.
5. Hoggart, C.J., Chadeau-Hyam, M., Clark, T.G., Lampariello, R., Whittaker, J.C., Iorio, M.D., and Balding, D.J. (2007). Sequence-Level Population Simulations Over Large Genomic Regions. Genetics *177*, 1725-1731.
6. Gravel, S., Henn, B.M., Gutenkunst, R.N., Indap, A.R., Marth, G.T., Clark, A.G., Yu, F., Gibbs, R.A., Project, T.G., and Bustamante, C.D. (2011). Demographic history and rare allele sharing among human populations. Proc Natl Acad Sci U S A *108*, 11983-11988.
7. Skoglund, P., and Mathieson, I. (2018). Ancient Genomics of Modern Humans: The First Decade. Annu Rev Genomics Hum Genet *19*, 381-404.
8. Allentoft, M.E., Sikora, M., Sjögren, K.-G., Rasmussen, S., Rasmussen, M., Stenderup, J., Damgaard, P.B., Schroeder, H., Ahlström, T., Vinner, L., et al. (2015). Population genomics of Bronze Age Eurasia. Nature *522*, 167-172.
9. Kılınç, G.M., Omrak, A., Özer, F., Günther, T., Büyükkarakaya, A.M., Bıçakçı, E., Baird, D., Dönertaş, H.M., Ghalichi, A., Yaka, R., et al. (2016). The Demographic Development of the First Farmers in Anatolia. Curr Biol *26*, 2659-2666.
10. Mathieson, I., Alpaslan-Roodenberg, S., Posth, C., Szécsényi-Nagy, A., Rohland, N., Mallick, S., Olalde, I., Broomandkhoshbacht, N., Candilio, F., Cheronet, O., et al. (2018). The genomic history of southeastern Europe. Nature *555*, 197-203.
11. Mathieson, S., and Mathieson, I. (2018). FADS1 and the Timing of Human Adaptation to Agriculture. Mol Biol Evol *35*, 2957-2970.
12. Olalde, I., Brace, S., Allentoft, M.E., Armit, I., Kristiansen, K., Booth, T., Rohland, N., Mallick, S., Szécsényi-Nagy, A., Mittnik, A., et al. (2018). The Beaker phenomenon and the genomic transformation of northwest Europe. Nature *555*, 190-196.
13. Lazaridis, I., Patterson, N., Mittnik, A., Renaud, G., Mallick, S., Kirsanow, K., Sudmant, P.H., Schraiber, J.G., Castellano, S., Lipson, M., et al. (2014). Ancient human genomes suggest three ancestral populations for present-day Europeans. Nature *513*, 409-413.
14. Haak, W., Lazaridis, I., Patterson, N., Rohland, N., Mallick, S., Llamas, B., Brandt, G., Nordenfelt, S., Harney, E., Stewardson, K., et al. (2015). Massive migration from the steppe was a source for Indo-European languages in Europe. Nature *522*, 207-211.
15. Bergström, A., McCarthy, S.A., Hui, R., Almarri, M.A., Ayub, Q., Danecek, P., Chen, Y., Felkel, S., Hallast, P., Kamm, J., et al. (2020). Insights into human genetic variation and population history from 929 diverse genomes. Science *367*, eaay5012.
16. Beaumont, M.A. (2010). Approximate Bayesian Computation in Evolution and Ecology. Annu Rev Ecol Evol S *41*, 379-406.
17. Tavaré, S., Balding, D.J., Griffiths, R.C., and Donnelly, P. (1997). Inferring Coalescence Times From DNA Sequence Data. Genetics *145*, 505-518.
18. Beaumont, M.A., and Rannala, B. (2004). The Bayesian revolution in genetics. Nat Rev Genet *5*, 251-261.

19. Beaumont, M.A., Zhang, W., and Balding, D.J. (2002). Approximate Bayesian Computation in Population Genetics. Genetics *162*, 2025-2035.
20. Kerner, G., Ramirez-Alejo, N., Seeleuthner, Y., Yang, R., Ogishi, M., Cobat, A., Patin, E., Quintana-Murci, L., Boisson-Dupuis, S., Casanova, J.-L., et al. (2019). Homozygosity for TYK2 P1104A underlies tuberculosis in about 1% of patients in a cohort of European ancestry. Proc Natl Acad Sci U S A *116*, 10430-10434.
21. Boisson-Dupuis, S., Ramirez-Alejo, N., Li, Z., Patin, E., Rao, G., Kerner, G., Lim, C.K., Krementsov, D.N., Hernandez, N., Ma, C.S., et al. (2018). Tuberculosis and impaired IL-23–dependent IFN-γ immunity in humans homozygous for a common TYK2 missense variant. Sci Immunol *3*, eaau8714.
22. Bos, K.I., Harkins, K.M., Herbig, A., Coscolla, M., Weber, N., Comas, I., Forrest, S.A., Bryant, J.M., Harris, S.R., Schuenemann, V.J., et al. (2014). Pre-Columbian mycobacterial genomes reveal seals as a source of New World human tuberculosis. Nature *514*, 494-497.
23. Sabin, S., Herbig, A., Vagene, A.J., Ahlstrom, T., Bozovic, G., Arcini, C., Kuhnert, D., and Bos, K.I. (2020). A seventeenth-century Mycobacterium tuberculosis genome supports a Neolithic emergence of the Mycobacterium tuberculosis complex. Genome Biol *21*, 201.
24. Durand, J.D. (1974). Historical Estimates of World Population: An Evaluation.(University of Pennsylvania).
25. Dubos, R.J., and Dubos, J. (1987). The White Plague: Tuberculosis, Man, and Society.(Rutgers University Press).
26. Francois, O., and Jay, F. (2020). Factor analysis of ancient population genomic samples. Nat Commun *11*, 4661.
27. Gunther, T., and Nettelblad, C. (2019). The presence and impact of reference bias on population genomic studies of prehistoric human populations. PLoS Genet *15*, e1008302.
